# Scene complexity modulates degree of feedback activity during object detection in natural scenes

**DOI:** 10.1371/journal.pcbi.1006690

**Published:** 2018-12-31

**Authors:** Iris I. A. Groen, Sara Jahfari, Noor Seijdel, Sennay Ghebreab, Victor A. F. Lamme, H. Steven Scholte

**Affiliations:** 1 New York University, Department of Psychology, New York, New York, United States of America; 2 Spinoza Centre for Neuroimaging, Royal Netherlands Academy of Arts and Sciences (KNAW), Amsterdam, The Netherlands; 3 University of Amsterdam, Department of Psychology, Section Brain and Cognition, Amsterdam, The Netherlands; 4 University of Amsterdam, Department of Informatics, Intelligent Systems Lab, Amsterdam, The Netherlands; Harvard University, UNITED STATES

## Abstract

Selective brain responses to objects arise within a few hundreds of milliseconds of neural processing, suggesting that visual object recognition is mediated by rapid feed-forward activations. Yet disruption of neural responses in early visual cortex beyond feed-forward processing stages affects object recognition performance. Here, we unite these discrepant findings by reporting that object recognition involves enhanced feedback activity (recurrent processing within early visual cortex) when target objects are embedded in natural scenes that are characterized by high complexity. Human participants performed an animal target detection task on natural scenes with low, medium or high complexity as determined by a computational model of low-level contrast statistics. Three converging lines of evidence indicate that feedback was selectively enhanced for high complexity scenes. First, functional magnetic resonance imaging (fMRI) activity in early visual cortex (V1) was enhanced for target objects in scenes with high, but not low or medium complexity. Second, event-related potentials (ERPs) evoked by target objects were selectively enhanced at feedback stages of visual processing (from ~220 ms onwards) for high complexity scenes only. Third, behavioral performance for high complexity scenes deteriorated when participants were pressed for time and thus less able to incorporate the feedback activity. Modeling of the reaction time distributions using drift diffusion revealed that object information accumulated more slowly for high complexity scenes, with evidence accumulation being coupled to trial-to-trial variation in the EEG feedback response. Together, these results suggest that while feed-forward activity may suffice to recognize isolated objects, the brain employs recurrent processing more adaptively in naturalistic settings, using minimal feedback for simple scenes and increasing feedback for complex scenes.

## Author summary

How much neural processing is required to detect objects of interest in natural scenes? The speed and efficiency of object recognition suggests that fast feed-forward buildup of perceptual activity is sufficient. However, there is also evidence that disruption of visual processing beyond feed-forward stages leads to decreased object detection performance. Our study resolves this discrepancy by identifying scene complexity as a driver of recurrent activity. We show that recurrent activity is enhanced for complex, cluttered scenes compared to simple, well-organized scenes. Moreover, recurrent activity reflects the amount of accumulated evidence for target object presence. These findings elucidate the neural processes underlying perceptual decision-making by demonstrating that the brain dynamically directs neural resources based on the complexity of real-world visual inputs.

## Introduction

Object recognition is often regarded as a task that is solved in the first wave of visual processing [1]. The human brain indeed recognizes objects at astonishing speed, with single neurons exhibiting object-selectivity from 100 ms after stimulus onset [2], and global brain signals diverging within 100–200 ms [3,4]. Furthermore, hierarchical feed-forward models can emulate human object recognition performance [5,6], and neural representations in human and non-human primate brains match those in feed-forward neural networks [7–9].

However, the visual system is not a strict feed-forward hierarchy: it also contains an abundance of feedback connections [10–13]. Visual response modulations that occur within visual cortex after the initial feed-forward sweep has passed (i.e. beyond ~150 ms after stimulus onset) are thought to reflect recurrent interactions (‘feedback’) between e.g. V1-IT that aid segmentation of figures from backgrounds [14–19] and perceptual grouping [20]. One way in which feedback is thought to facilitate object recognition is through *visual routines* such as curve tracing and texture segmentation, to integrate line segments and other low-level features encoded in early visual areas [21–23]. Supporting this model of visual processing, transcranial stimulation evidence shows that detection of target objects in natural scenes deteriorates when neural activity in early visual cortex is disrupted not just at feed-forward processing stages (e.g., 100 ms after stimulus onset), but also at feedback stages (e.g., 220 ms after stimulus onset [24,25]).

**Fig 1:**
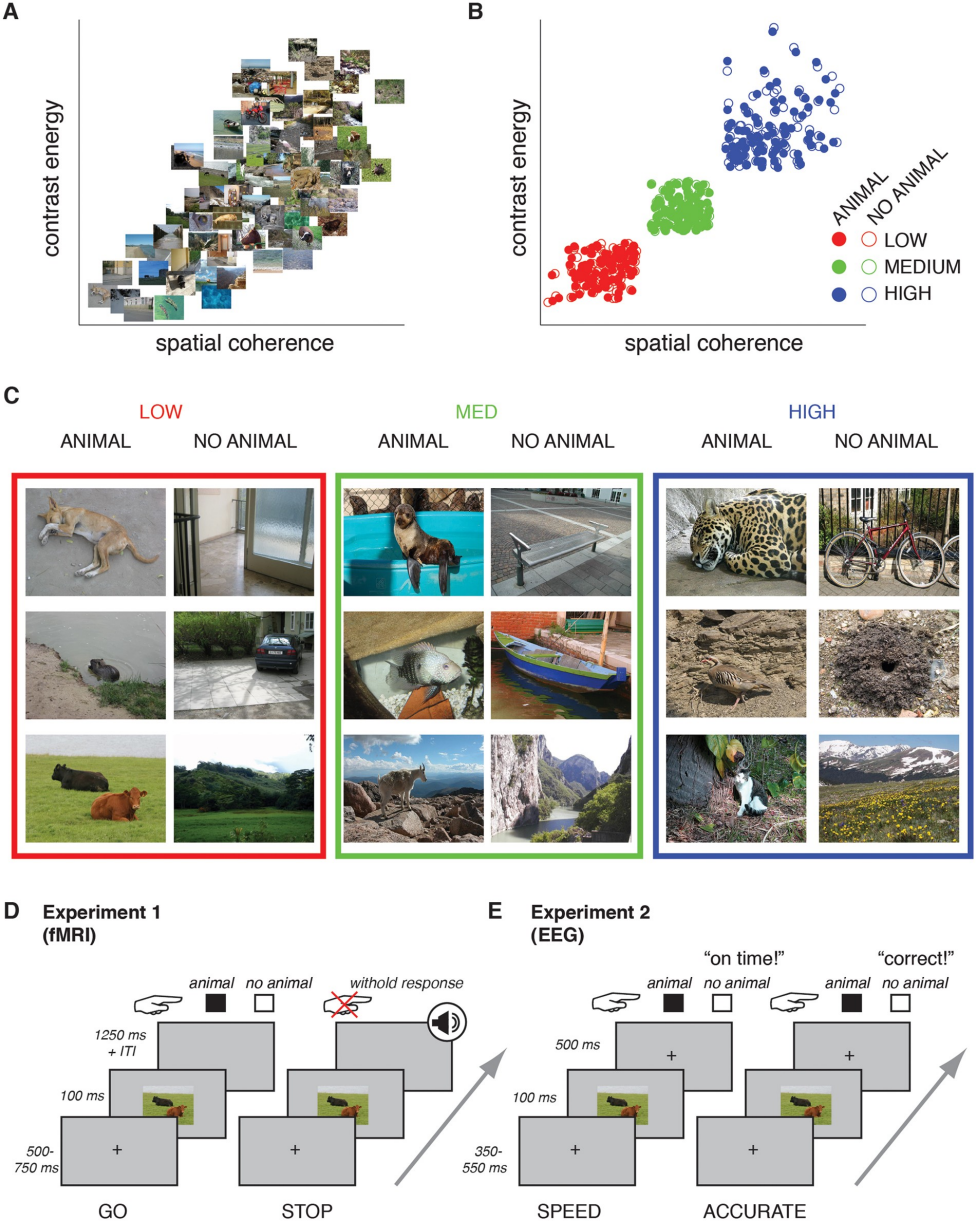
Stimuli and experimental paradigms. **A)** Stimulus space described by two parameters derived from the distribution of local contrast, contrast energy (CE) and spatial coherence (SC). In this space, simple images containing one or a few easily segmentable objects are on the lower left, while complex images with a high degree of fragmentation are on the upper right. Thumbnails show 100 images (50 animal, 50 non-animal) randomly drawn from the larger image set from which the stimuli were selected. **B)** Image statistics of the stimuli: each point represents a scene sampled from the image space described in A). Scenes had either low (red), medium (green) or high (blue) CE and SC values. Within these conditions, CE and SC values were matched between scenes with target objects (animals: “A”, filled dots) and without target objects (non-animals: “NA”, open dots). **C)** Exemplars from each stimulus complexity condition. **D)** Experimental design of Experiment 1 (fMRI). On GO trials, subjects indicated whether the scene contained a target object or not. On STOP trials, an auditory signal followed stimulus presentation after a variable inter-trial-interval (ITI), signaling that subjects had to withhold their response. Only GO trials were analyzed. **E)** Experimental design of Experiment 2 (EEG). Participants were instructed to respond as fast as possible on speed trials and as accurate as possible on accuracy trials and received feedback on each trial.

How can we reconcile the speed of object recognition with an important role for feedback? Two lines of evidence suggest that feedback may be employed *adaptively* depending on the complexity of the visual input. First, computer simulations indicate that disrupting feedback activity has stronger effects for occluded or degraded target objects [26]. Second, backward masking, which interrupts recurrent processing [27–29], has weaker effects for scenes with target objects that are “easily segregated” compared to “more demanding backgrounds”, as assessed through behavioral ratings of independent observers [30]. But how does the visual system determine the complexity of a visual input scene? Computer vision and scene perception research shows that computational summary statistics of low-level image features are diagnostic of scene complexity [31–33]. For example, contrast distributions can be summarized by two statistics that reflect a scene’s contrast energy (CE, average contrast) and spatial coherence (SC, variability in contrast across the scene) [34,35]. Computing these statistics for a large set of scenes results in a two-dimensional space in which sparse scenes with just a few scene elements separate from complex scenes with a lot of clutter and a high degree of fragmentation (Fig 1*A*). Since CE and SC appear to provide information about the ‘segmentability’ of a scene, we hypothesized that the visual system computes these scene statistics as a measure of overall scene complexity, with the goal to determine a need for enhanced visual processing mediated by feedback.

Importantly, for this hypothesis to be biologically realistic, CE and SC need to be a) plausibly computable in the visual system and b) available early in visual processing. The contrast distribution of a scene can in theory be deduced from the population response of contrast-sensitive neurons, e.g. neurons in early visual areas such as LGN and V1, which respond to local scene elements (edges). Since these responses constitute the first stage of the feedforward processing cascade, this information can be made available very early on in visual processing through a ‘read-out’ of the early population response across the visual scene within the feed-forward sweep. Consistent with this idea, CE and SC values computed from simulated early visual contrast responses [35,36] have been shown to modulate the magnitude of single-trial evoked responses to natural scenes as early as 100 ms after stimulus onset [35,37–42].

Here, we tested whether scene complexity predicts the degree of feedback activity by measuring brain responses while participants performed a target object detection task in scenes that were systematically sampled to contain either low, medium or high CE and SC values (see Fig 1*B* and 1*C*). First, we measured whole-brain fMRI responses to target objects in scenes with low, medium or high complexity (Experiment 1). Next, we measured EEG responses to the same stimuli to examine the time-course of visually evoked activity (Experiment 2). Importantly, complexity was matched for target and non-target scenes *within* each complexity condition, allowing us to disambiguate any feedforward response differences due to scene complexity from subsequent feedback modulations by examining differential responses to target and non-targets. In addition, scene complexity was varied on a trial-by-trial basis, such that participants could not form an expectation of scene complexity beforehand, allowing us to measure responses with unbiased feed-forward processing (i.e. without a difference in top-down task set or attentional state).

Together, these experiments show that successful detection of target objects in high, but not low or medium complexity scenes is associated with enhanced activity in early visual areas (in fMRI) which emerges at feedback time-points in visual processing (in EEG). Moreover, behavioral performance for high complexity scenes was associated with decreased accuracy and slower response times, reflecting a slower rate of evidence accumulation as formalized by the drift rate parameter in the drift-diffusion model [43–45]. In addition, trial-by-trial variations in drift rate within the high complexity condition were coupled to EEG feedback responses on those trials. Together, these results demonstrate a contribution of feedback to object detection in complex natural scenes.

## Results

### Experiment 1 (fMRI)

#### Behavior

**Fig 2:**
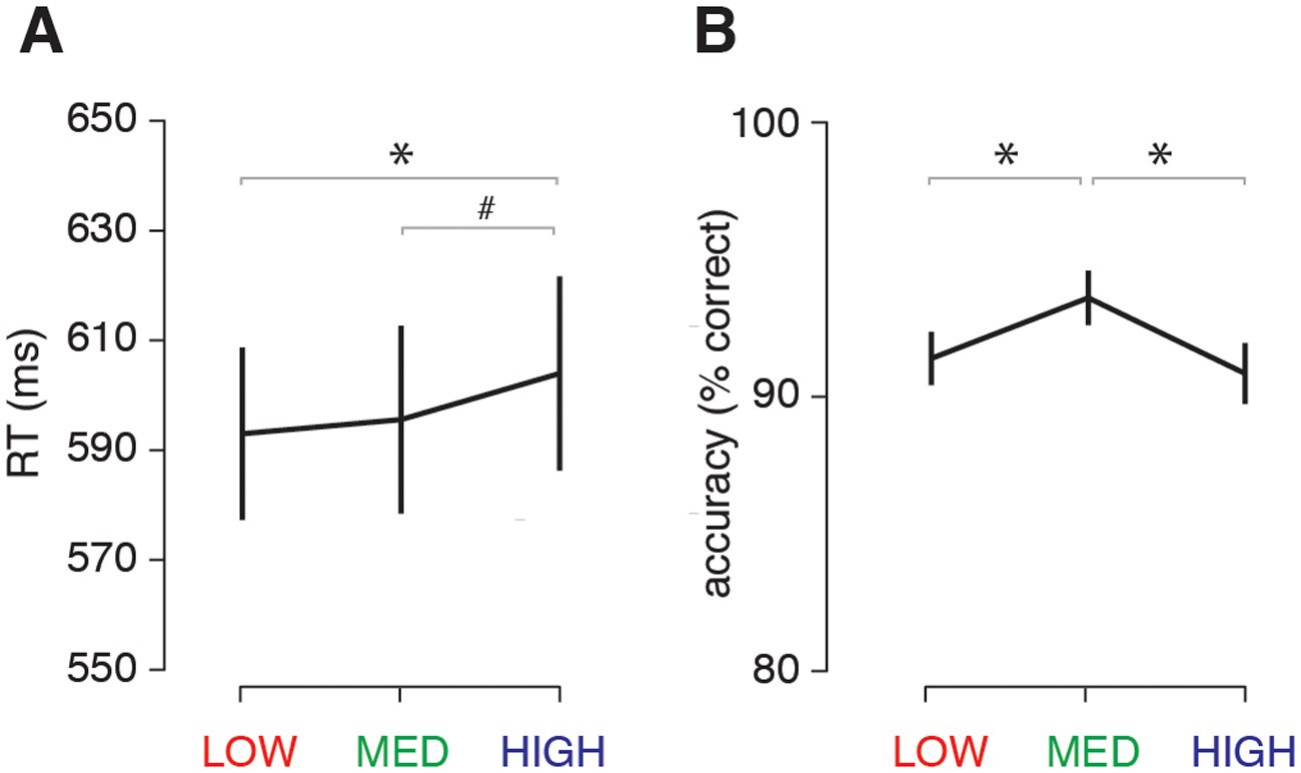
Behavioral results of the fMRI experiment (Experiment 1). **A)** Average reaction times (RT) for the animal/non-animal categorization task per condition. **B)** Accuracy (percentage correct) per condition. Horizontal black lines indicate the statistical outcome of one-way repeated-measures ANOVAs; gray lines indicate the results of pairwise tests corrected for multiple comparisons using a Sidák correction. Error bars represent S.E.M. * = p < 0.05, # = p < 0.10.

In Experiment 1, participants viewed images of real-world scenes with low, medium or high complexity in the MRI scanner. On each trial, they indicated via a button press whether the scene contained a target object (animal) or not (Fig 1D). This detection task was embedded in a stop-signal paradigm, but for the purpose of this study, only the trials without a stop signal (‘go trials’) were analyzed (see Materials and methods). Behavioral performance for each condition is presented in Fig 2. Reaction times were found to increase gradually with scene complexity (F(2,44) = 4.96, p = 0.011, η^2^ = 0.18). Planned post-hoc comparisons showed that RTs increased for the HIGH complexity condition compared to the LOW (t(22) = 2.7, p(Sidák-corrected) = 0.035), and the MEDIUM condition (t(22) = 2.5, p(Sidák-corrected) = 0.057), with no significant difference between the LOW and MEDIUM conditions (t(22) = 0.7, p(Sidák-corrected) = 0.85; Fig 2A).

Response accuracy was also modulated by scene complexity (F(2,44) = 8.37, p = 0.001, η^2^ = 0.28). Post-hoc comparisons showed the best performance for the MEDIUM condition compared to the LOW (t(22) = 3.5, p(Sidák-corrected) = 0.007) and HIGH condition (t(22) = 3.2, p(Sidák-corrected) = 0.012), with no differences between the LOW and HIGH conditions (t(22) = 0.8, p(Sidák-corrected) = 0.77; Fig 2*B*).

Together, these results indicate that the high scene complexity leads to significant slowing in object detection. Although response accuracy in both the LOW and HIGH condition was worse than for the MEDIUM condition, responses were only slowed for HIGH complexity scenes, suggesting that the HIGH condition was experienced as most difficult.

#### fMRI results: Whole brain analysis

**Fig 3:**
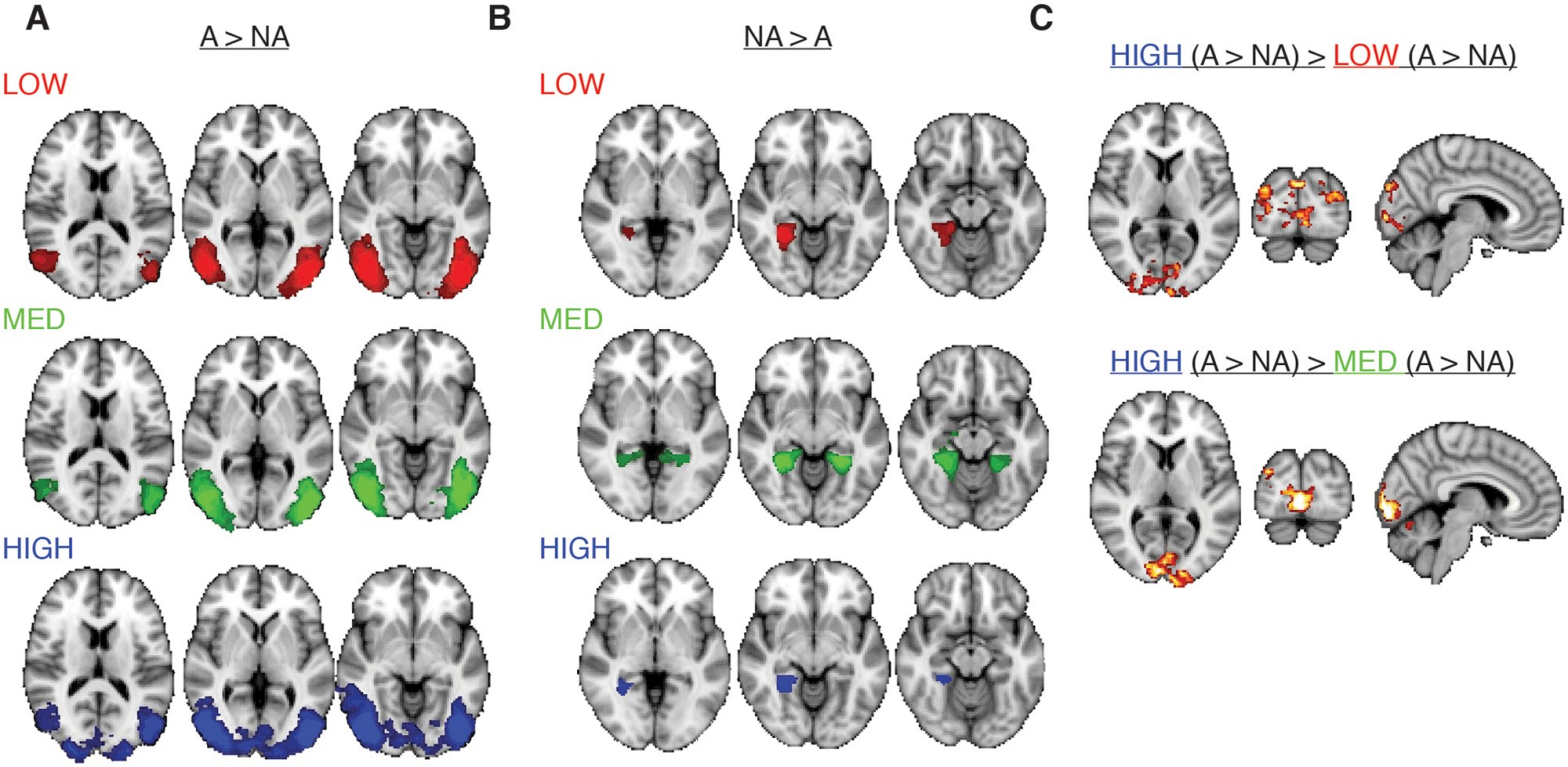
Whole brain fMRI results. **A)** Statistical parametric maps for the animal (A) > non-animal (NA) contrast for each condition. From left to right, MNI coordinates for each transversal slice are z = [10, 2, –6]. **B)** Statistical parametric maps for the non-animal > animal contrast for each condition. From left to right, MNI coordinates are z = [–2, –8, –14]. **C)** Differences in the differential animal vs. non-animal activity between conditions. MNI-coordinates: [x = 6, y = -88, z = 6]. Maps were cluster-corrected and thresholded at z = 2.3. Color scales range from z = 2.5 to 5 (A and B) and z = 2.3 to 3.5 (C).

Whole-brain comparisons of target-present (animal) vs. target-absent (non-animal) scenes revealed significant clusters in lateral and ventral high-level visual cortex (Fig 3). For the animal > non-animal contrast (Fig 3*A*), bilateral clusters overlying lateral occipital cortex were found in all conditions. Critically, in the HIGH condition, additional differential activity was found in low-level visual areas. Indeed, contrasting these statistical maps between conditions revealed a large cluster in several early visual areas (Fig 3*B*) and smaller clusters in inferior parietal regions. For the non-animal > animal contrast (Fig 3*C*), bilateral clusters in parahippocampal cortex were found in the MEDIUM condition, while in the LOW and HIGH condition, only right-lateralized clusters survived whole-brain cluster-correction. Contrasting the difference between non-animal > animal scenes between conditions resulted in no significant clusters. Cluster coordinates for all contrasts are reported in Table 1.

**Table 1:** Whole brain fMRI analysis cluster coordinates for the significant contrasts. COG = center of gravity, p = p-value, lat. = lateral, occ. = occipital, temp. = temporal, inf. = inferior, post. = posterior, L = left, R = right. Coordinates are reported in MNI space. Areas included in the clusters were determined by determining overlap of local maxima within clusters with the probability maps of the Juelich Histological Atlas and the Harvard-Oxford Cortical Structural Atlas implemented in FSL. Note that for the contrasts HIGH (animal—non-animal), MED (non-animal—animal) and [HIGH (animal-non-animal)—MED (animal- non-animal)], the clusters are bilateral, forming one cluster across hemispheres (see also Fig 3).

contrast	size (mm^3^)	log10 (p)	x	y	z	areas included
animal > non-animal						
LOW	43656	15.6	45	-66	-9	V4, V5, lat. occ. cortex. inf. division, inf. temp. gyrus, temp. occ. fusiform. gyrus (R)
	43472	15.6	-41	-71	-8	V4, V5, lat. occ. cortex. inf. division, inf. temp. gyrus, temp. occ. fusiform. gyrus (L)
MED	39296	15.7	45	-70	-6	V4, V3v, lat. occ. cortex. inf. division, inf. temp. gyrus, temp. occ. fusiform. gyrus, occ. fusiform gyrus (R)
	39976	15.9	-42	-74	-6	V4, V5, lat. occ. cortex. inf. division, inf. temp. gyrus, temp. occ. fusiform. gyrus. (L)
HIGH	142168	36.1	3	-74	-3	V1, V2, V3, V4, V5, lat. occ. cortex. inf. division, inf. temp. gyrus, temp. occ. fusiform. gyrus. (L+R).
non-animal > animal						
LOW	4585	1.96	27	-45	-9	parahippocampal gyrus post. div., lingual gyrus, temp. occ fusiform cortex (R)
MED	29872	5.78	4	49	2	parahippocampal gyrus post. div., lingual gyrus, temp. occ. fusiform cortex, supracalcarine cortex, precuneus (L+R)
HIGH	4392	1.81	26	-47	-1	parahippocampal gyrus post. div. lingual gyrus, temp. occ. fusiform cortex. supracalcarine cortex, precuneus (R)
animal > non-animal, between conditions						
HIGH > MED	14536	6.32	7	-85	-2	V1, V2, V3V, V4, occ. fusiform gyrus, lingual gyrus (R).
	7992	3.61	-15	-95	19	V1, V2, V3V, inf. parietal lobule, occ. fusiform gyrus, lingual gyrus (R)
HIGH > LOW	21016	8.94	2	-90	4	V1, V2, V3V, inf. parietal lobule, lingual gyrus, (L+R)

#### fMRI results: ROI analysis

Following the whole brain analysis, the difference in BOLD activity for animal vs. non-animal scenes in each condition was computed in four *a priori*, independently defined regions of interest and compared across conditions using repeated-measures ANOVAs. In line with the whole brain results, scene complexity was found to modulate activity in early visual cortex (V1: (F(2,44) = 4.9, p = 0.01, η^2^ = 0.18; Fig 3*A*). Planned post-hoc comparisons indicated that this effect was driven by increased activity for animal vs. non-animal scenes in the HIGH compared with the MEDIUM condition (t(22) = 2.6, p(Sidák-corrected) = 0.05) and a trend towards increased differential activity in the HIGH versus the LOW condition (t(22) = 2.3, p(Sidák-corrected) = 0.09), with no difference between LOW and MEDIUM conditions (t(22) = 1.2, p(Sidák-corrected) = 0.54).

In addition, one-sample t-tests conducted for each of the three conditions indicated that the differential activity in V1 significantly deviated from zero only in the HIGH condition (HIGH: t(22) = 2.8, p(Sidák-corrected) = 0.03; LOW: t(22) = 0.19, p(Sidák-corrected) = 0.99; MEDIUM: t(22) = -1.28, p(Sidák-corrected) = 0.52).

**Fig 4:**
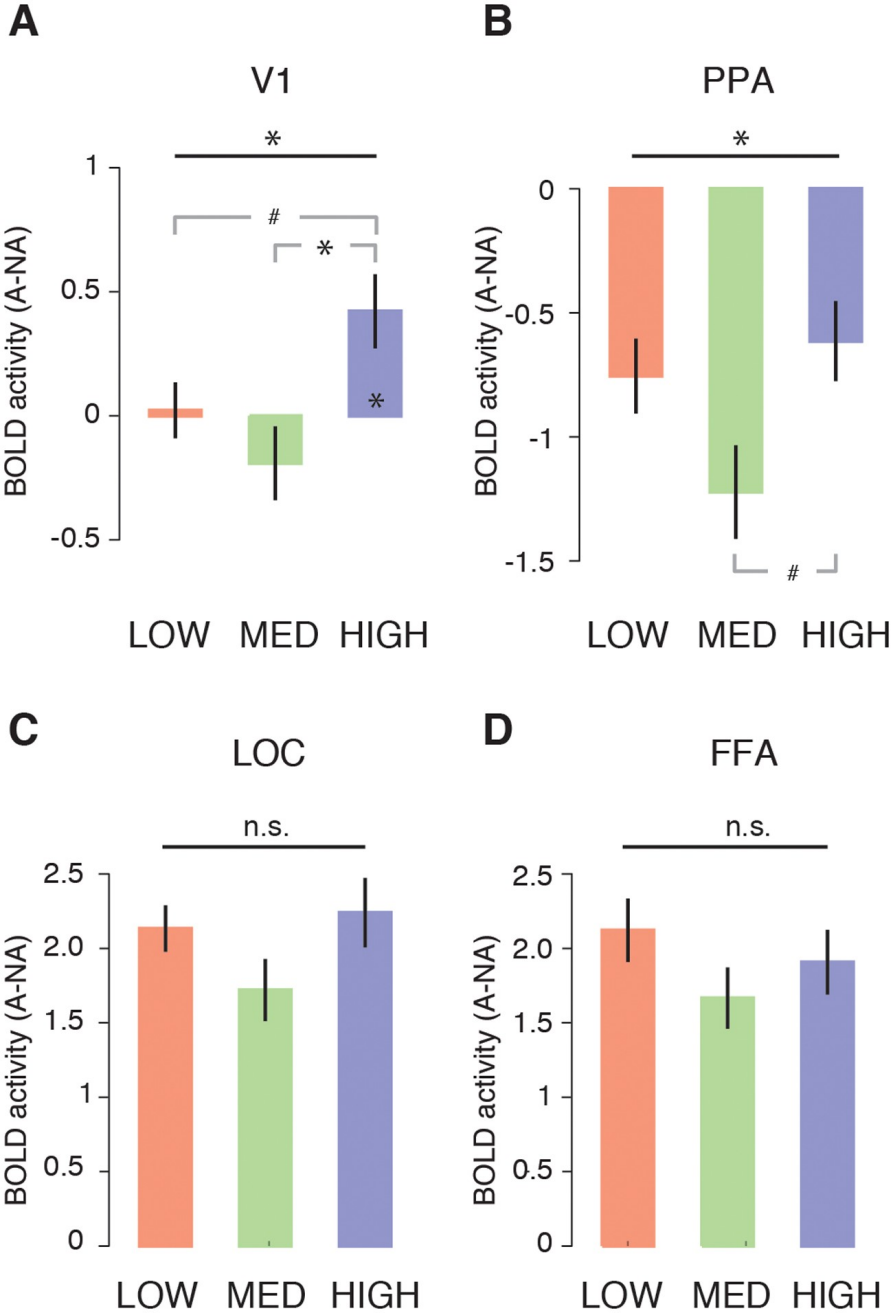
fMRI ROI analysis results. Differential activity for animal vs. non-animal scenes for each condition in **A)** V1, **B)** PPA, **C)** LOC and **D)** FFA. Horizontal black lines indicate the statistical outcome of repeated-measures ANOVAs; gray lines indicate the results of pairwise tests corrected for multiple comparisons using a Sidák correction. * = p < 0.05, # = p < 0.10. Error bars represent S.E.M.

We also observed a main effect of scene complexity in place-selective PPA (F(2,44) = 3.6, p = 0.04, η^2^ = 0.14 (Fig 4*B*), which exhibited stronger responses for non-animal than animal scenes. Planned post-hoc comparisons indicated a trend towards less differential activity for the HIGH compared with the MEDIUM (t(22) = 2.4, p(Sidák-corrected) = 0.07), but not the LOW condition (t(22) = 1.9, p(Sidák-corrected) = 0.19), and no difference between the LOW and HIGH conditions (t t(22) = 0.7, p(Sidák-corrected) = 0.89). In contrast, object-selective LOC and face-selective FFA both responded more positively to scenes with animals than to scenes without animals (Fig 4*C* and 4*D*), but these responses did not differ across conditions (FFA: F(2,44) = 1.7, p = 0.19, η^2^ = 0.07; LOC: F(2,42) = 1.9, p = 0.14, η^2^ = 0.08).

#### Summary Experiment 1

In sum, behavioral detection of target objects (animals) in natural scenes was slower and less accurate for scenes of increased complexity. The presence of a target object was associated with an enhanced fMRI response in known object- and face-selective regions, but these responses were not significantly modulated by scene complexity. In contrast, scene complexity modulated activity levels in V1 and scene-selective PPA. In PPA, differential activity related to target-presence was least pronounced for the high complexity scenes compared to the medium complexity scenes, while V1 activity was only modulated by target presence for high complexity scenes. Taken together, these results indicate that scenes with high complexity induced the least difference in PPA while giving rise to additional differential activity in early visual areas.

These fMRI results suggest that information encoded in early visual cortex may be selectively recruited during target detection for the most difficult, high complexity scenes. Since the scenes were carefully matched in CE and SC *within* complexity levels, it is unlikely that this activity was driven by differences in low-level properties between target and non-target scenes evoked by feed-forward processing. Therefore, we hypothesized that the differential V1 early visual activity results from increased recurrent activity elicited by a need for additional visual processing to detect the target object in high complexity scenes.

However, two limitations preclude a strong confirmation of this hypothesis. First, due to the poor temporal resolution of the fMRI signal, we cannot exclude the possibility that early visual activity differences were present as a result of other differences between the target and non-target scenes than those captured by CE and SC. Second, the categorization task was embedded in a stop-signal paradigm (see Materials and methods). Although all results reported above were based on GO trials only (i.e. omitting all trials in which a stop signal was presented), the observed effects could be specific to, or amplified by, an expectation of a stop instruction, and might not generalize to a task in which participants are never asked to withhold their response.

To overcome these two limitations, we conducted a second experiment, in which we recorded EEG while participants performed the same animal detection task on the same set of scenes without any stop-signal manipulation. First, to test our hypothesis that the activity differences in early visual areas were feedback-related, we computed event-related potentials (ERPs) for target and non-target scenes separately in each condition, to infer when in time neural responses started to differ on electrodes overlying early visual cortex. Since scenes were matched in image statistics, we predicted that there would be no differences between animal and non-animal scenes in feedforward responses, i.e. before ~150 ms after stimulus onset, after which ERP responses are thought to start reflecting recurrent activity [23,28,46]. Second, because behavioral performance for high complexity scenes in Experiment 1 was characterized by slowed RTs with no substantial improvements in detection accuracy, the behavioral task in the EEG experiment was divided into blocks with a task instruction to emphasize either ‘speed’ or ‘accuracy’ (Fig 1*E*). We hypothesized that if target detection in high complexity scenes was indeed associated with increased feedback, performance in the HIGH condition should be most affected for speeded trials, in which extensive visual processing is limited by time constraints.

### Experiment 2 (EEG)

#### Behavior and HDDM parameters

**Fig 5:**
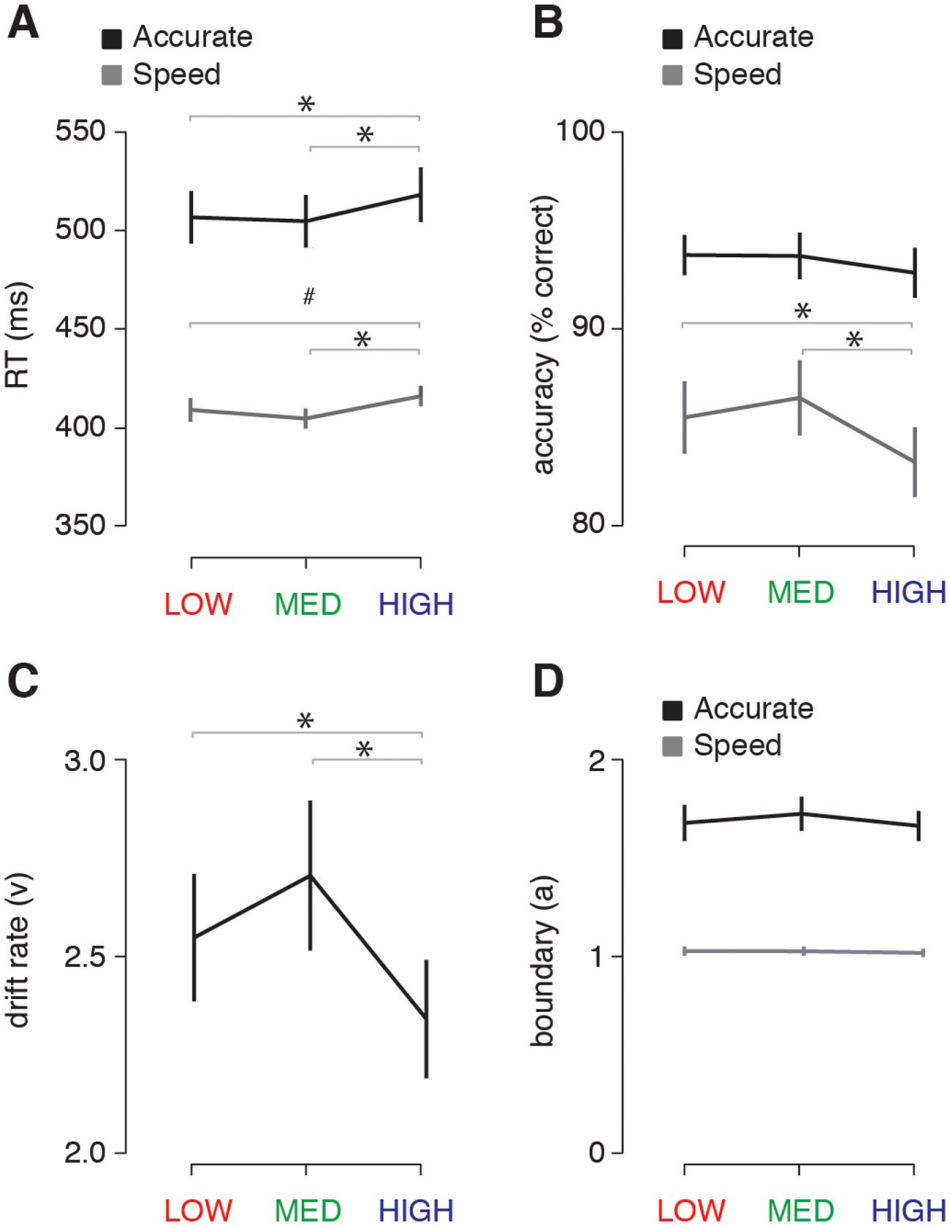
Behavioral and HDDM modeling results of the EEG experiment (Experiment 2). **A)** Average reaction times (RT) per condition and task instruction. **B)** Accuracy (percentage correct) per condition and task instruction. **C)** Estimates of drift rates per condition, indicating slower evidence accumulation for high complexity scenes. **D)** Estimates of the amount of evidence required (boundary) per condition and task instruction. Gray lines indicate the results of pairwise post-hoc tests between conditions corrected for multiple comparisons using a Sidák correction. Error bars represent S.E.M. * = p < 0.05.

Fig 5 shows an overview of the behavioral results in Experiment 2. Analysis of the reaction times showed a significant main effect for instruction (speed or accurate; F(1,25) = 87.6, p < 0.001, η^2par^ = 0.78), and scene complexity (LOW, MEDIUM, or HIGH; F(2,50) = 29.1, p < 0.001, η^2par^ = 0.54). As expected, participants responded faster under speed instructions, and response times were again longest for the high complexity scenes. Planned post-hoc comparisons further showed that responses were significantly slower for high compared with medium complexity scenes under both instructions (speed: t(25) = 5.0, p < 0.001; accurate: t(25) = 5.1, p = < 0.001; all Sidák-corrected; Fig 5*A*). Compared to low complexity scenes, responses in the high complexity condition were significantly slower for accurate task instructions (t(25) = 4.9, p > 0.001), while showing a similar trend for the speed instruction (t(25) = 2.7, p = 0.065; all Sidák-corrected). Response times did not differ between low and medium conditions (speed: t(25) = 2.4, p = 0.13; accurate: t(25) = 0.72, p = 0.98; all Sidák-corrected).

Accuracy scores showed a significant interaction between scene complexity and task-instruction (F(2,50) = 5.6, p = 0.002, η^2par^ = 0.18). That is, detection accuracy was selectively decreased for highly complex scenes when participants were motivated to respond as fast as possible (HIGH vs. LOW, t(25) = 3.2, p = 0.024; HIGH vs. MEDIUM, t(25) = 4.4, p = 0.001; LOW vs. MEDIUM, t(25) = 1.98, p = 0.38, all Sidák-corrected). This effect was not found for trials in which participants were asked to be as accurate as possible (HIGH vs. LOW; t(25) = 1.5, p = 0.60; HIGH vs. MEDIUM, t(25) = 1.8, p = 0.42; LOW vs. MEDIUM: t(25) = 0.1, p = 0.99, all Sidák-corrected; Fig 5*B*).

In summary, these results indicate increased reaction times for high complexity scenes both when participants are pressed for time (speed trials), or when asked to make the most accurate response (accurate trials). However, detection accuracy is decreased only when participants are pressed for time (Fig 5B). This suggests that for high complexity scenes, visual information processing might be too slow to benefit detection, especially when participants are motivated to respond quickly and thus should have lower evidence requirements in comparison to accurate instruction trials.

To formally support this assumption, we modeled the RT and detection accuracy data with a hierarchical implementation of the drift diffusion model (HDDM), which details how latent parameters describing the speed of sensory information accumulation (drift rate) and evidence requirements (boundary) each contribute to reaction times and response accuracy [44,45,47]. We found that drift rate was signficantly modulated across the three scene complexity conditions (F(2,50) = 12.5, p<0.001, η^2par^ = 0.33), with the slowest rate of information accumulation for high complexity scenes (HIGH vs. LOW; t(25) = 2.97, p = 0.02; HIGH vs. MEDIUM, t(25) = 5.04, p < 0.001; LOW vs. MEDIUM: t(25) = 2.05, p = 0.15, all Sidák-corrected; Fig 5*C*). In contrast, the boundary only varied as a function of task instructions (F(1,25) = 93.5, p<0.001, η^2par^ = 0.79), and was not modulated by scene complexity (F(2,50) = 1.3, p = 0.22, η^2par^ = 0.05), and there was no interaction (F(2,50) = 2.5, p = 0.10, η^2par^ = 0.09; Fig 5*D*). This suggests that RTs and error rates are increased in the HIGH condition because information accumulation is slowed, but not because the requirements to reach a decision are changed.

Together, the behavioral results of Experiment 2 again indicated increased reaction times for high complexity scenes relative to less complex scenes. In addition, they revealed a slower rate of information processing (drift rate) for high complexity scenes, resulting in a selective decrease in performance when the decision is speeded.

#### Grand-average ERP results

**Fig 6:**
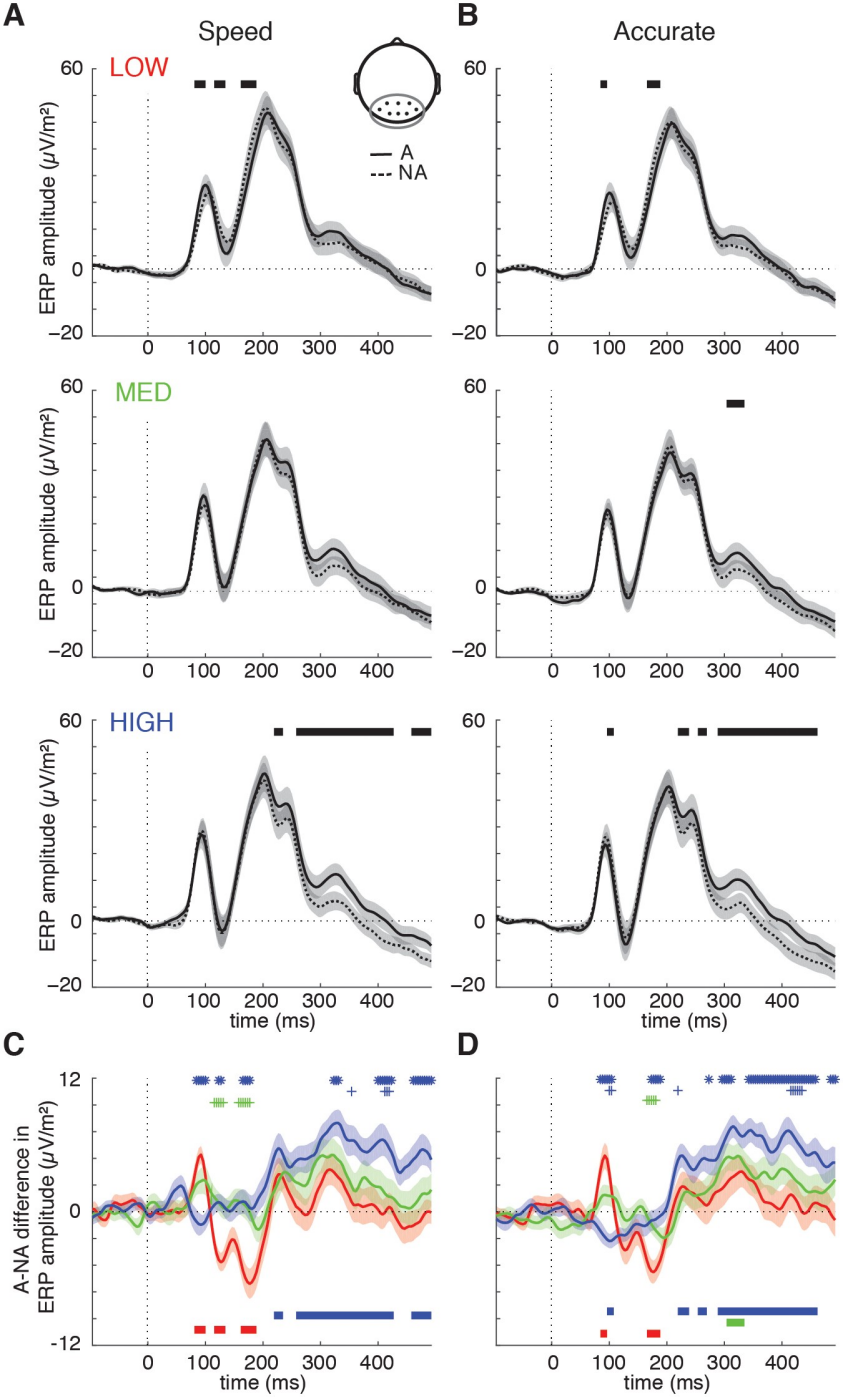
ERP results from the EEG experiment (Experiment 2). Average ERP amplitude for animal (solid lines) and non-animal scenes (dashed lines) per condition for an occipital-peri-occipital cluster of EEG channels (Oz, POz, O1, O2, PO3, PO4, PO7, PO8) for the **A)** speed and **B)** accuracy trials. Shaded regions indicate SEM across participants. Significant differences between animal and non-animal ERPs (FDR-corrected) are indicated with thick black lines (also in C/D). **C-D** Animal vs. non-animal difference wave for each condition overlaid and statistically compared (FDR-corrected) for the speed **A)** and accuracy **B)** trials. Thick lines at the bottom of the graphs indicate significant difference wave deflections from zero, and shadings indicate S.E.M. Symbol markers indicate significant differences in difference wave amplitude between conditions, blue asterisk: HIGH vs. LOW; blue plus: HIGH vs. MEDIUM; green plus: MEDIUM vs. LOW.

To investigate the time-course of object detection in visual cortex, evoked responses to the target and non-target scenes were pooled across a set of occipital and peri-occipital electrodes (see Materials and methods). The results in Fig 6 show that target and non-target ERPs before 150 ms were highly overlapping in all three conditions (although the ERPs diverged for a few time-points before 150 ms in the LOW condition). Critically, target and non-target ERPs in the HIGH condition only started to diverge from ~220 ms, suggesting a contribution of recurrent processing to target detection in high complexity scenes. These effects were consistent across the speed (Fig 6A) and accuracy trials (Fig 6B).

Statistical comparisons (FDR-corrected across all time-points, task-instructions and conditions) of the animal vs non-animal difference waves indicated that for speeded trials, the difference wave in the HIGH condition did not diverge from zero before prior to 220 ms (Fig 6*C*, blue solid line); in the accuracy trials, there was a brief interval of significant deflection before 200 ms (Fig 6*D*), but this reflected a reversed difference wave compared to the other two conditions. Beyond 220 ms, however, this pattern reversed: direct comparison of the difference wave across conditions indicated that the difference wave for the HIGH condition after 220 ms was significantly enhanced compared to the LOW (blue asterisks; top) and MEDIUM (blue plusses; top) conditions.

To exclude the possibility that the differential effects in HIGH simply reflected the absence of any object information in non-target trials, we repeated these analyses while restricting the non-animal trials to only those images that contained large, non-animal distractor objects (vehicles, humans and man-made objects). The results of this analysis were qualitatively similar (see S1 Fig), suggesting that these differential responses were not driven by the absence of distractor objects for non-animal trials.

The deviating shape of the difference wave in the HIGH condition relative to the other two conditions has two important implications. First, the lack of a reliable early (< 150 ms) difference wave in the HIGH condition indicates that it is unlikely that the differential fMRI activity in early visual areas found in Experiment 1 was driven by uncontrolled low-level image properties. Such properties are expected to elicit a difference in feed-forward responses, and thus an early difference in ERP responses. Instead, the strongly enhanced ERP difference beyond 220 ms in the HIGH condition suggests this to be the electrophysiological correlate of the differences in the early visual fMRI response.

Second, the late onset of the target vs. non-target distinction for HIGH provides a potential neural explanation for the decreased behavioral performance for these scenes. As shown in Fig 5, high scene complexity was associated with slower reaction times and lower drift rates, as well as decreased accuracy during speeded trials, because participants commit to a choice with less evidence on those trials (lower boundary; Fig 5D). The ERP results suggest that in the HIGH condition, participants relied on differential activity emerging at recurrent stages of visual processing (beyond 220 ms), which may be less accessible under time pressure (speeded trials). Thus, these observations suggest the rate of evidence accumulation for highly complex scenes was slowed due to enhanced recurrent processing. We examined this relation more closely in the next section, by comparing ERPs and behavioral performance at the single-trial level.

#### Relating ERPs to behavior

**Fig 7:**
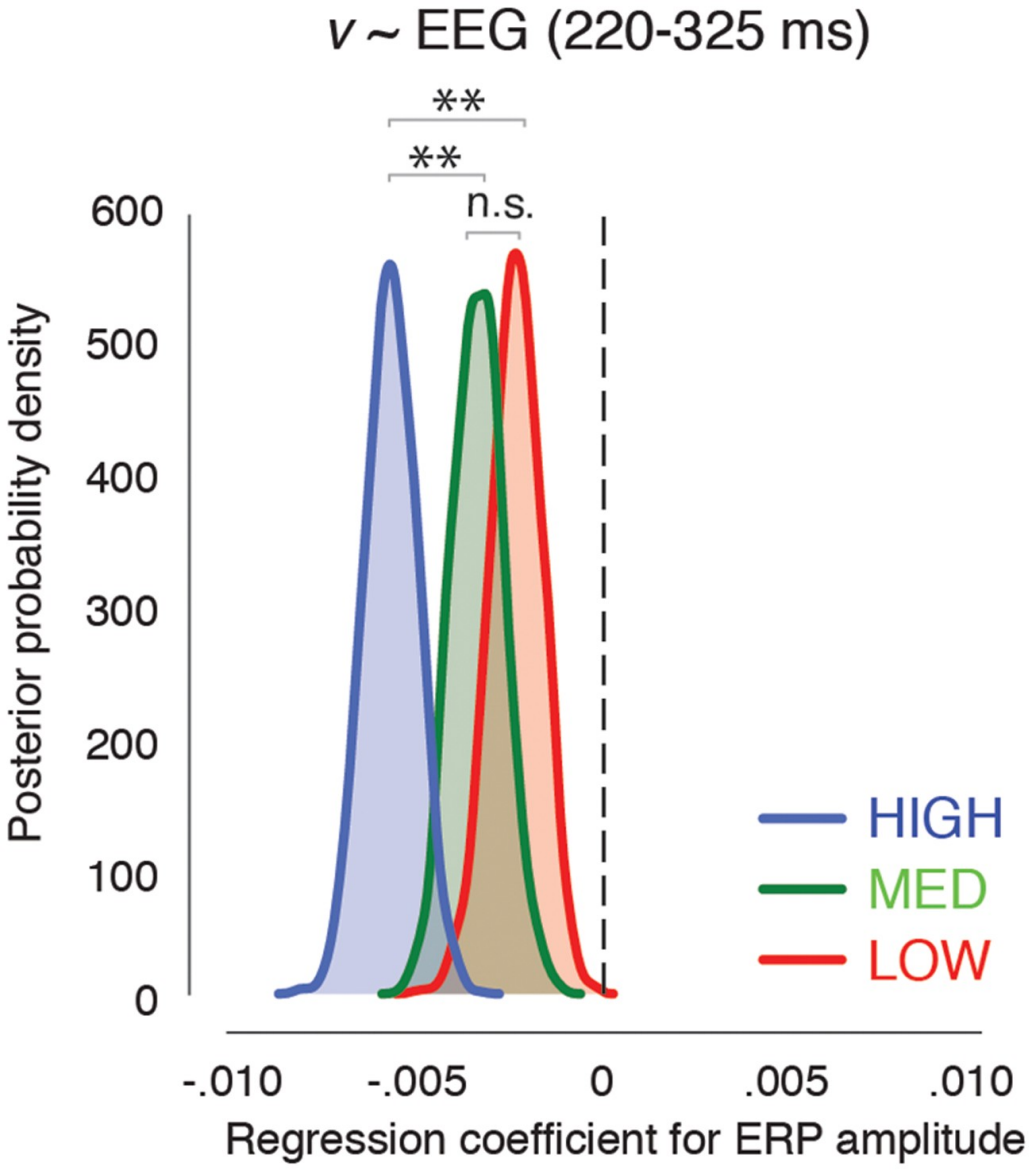
HDDM regression results. Posterior probabilities of the regression weight for drift rate (v) on the single-trial ERP amplitude averaged between 220–325 ms after stimulus onset for LOW, MED and HIGH complexity.

To assess whether EEG activity beyond 200 ms was indeed indicative of the accumulation of evidence for the target/non-target decision, we investigated the relation between ERP responses and drift rate using trial-by-trial analysis. We again fitted a HDDM model [45,48,49] to the RT distributions for target and non-target decisions in LOW, MED and HIGH, now including the single-trial ERP amplitude as a predictor (HDDM regression; see Materials and methods). Instead of estimating one drift rate per subject across trials, this model assumes the drift rate to vary on each trial according to a linear relation with their measured EEG activity. Since our behavioral analysis did not indicate an effect of task instruction on drift rates, we estimated this model across all trials (i.e., both speed and accurate) simultaneously. The results show that drift rates were indeed modulated significantly by trial-by-trial differences in late ERP amplitude (220–325 ms; Fig 7). The regression weight is negative, indicating that higher ERP amplitude in that interval reflects lower drift rates, consistent with our interpretation that slower evidence accumulation is associated with increased feedback processing. The regression weight was significantly different from zero in all conditions (all p = 0.0); however, it was significantly larger for HIGH relative to both LOW (p = 0.0) and MED (p = 0.0008) trials, whose regression weights did not differ from one another (p = 0.11). These results thus provide further support that late ERP activity (presumably reflecting recurrent interactions) reflects a relatively slowed accumulation of evidence for the target/non-target decision for scenes with high complexity.

#### Summary Experiment 2

The differential ERP response indicating target presence in the HIGH, but not the LOW and MEDIUM conditions emerged after 200 ms, suggesting that it does not reflect a feed-forward signal, but recurrent processing. Moreover, this feedback response reflected a slower rate of evidence accumulation at the single-trial level. While feedback activity was present for all trials (speeded or accurate), behavioral performance was only impaired during speeded trials. Because evidence requirements were lower during speed trials (Fig 5D), we reason that the feedback activity during those trials, while present, did not benefit task performance, because participants lacked sufficient time to incorporate the accumulating evidence into a behavioral response.

## Discussion

We investigated the influence of scene complexity on the detection of target objects in natural scenes using fMRI, EEG and behavior. Behaviorally, we observed prolonged detection times when participants viewed high compared with low or medium complexity scenes. Using fMRI, we found these slower responses to be accompanied by a selective increase in activity for target objects in early visual regions including V1, while target activity in higher-level object-selective cortex was not affected by scene complexity. EEG recordings showed that the differential response to targets and non-targets in early visual cortex arose after ~220 ms, suggesting it is likely driven by feedback/recurrent interactions, rather than feed-forward activity differences. While increased feedback for target objects in high complexity scenes was observed for both fast and accurate task instructions, behavioral performance only profited from this activity when the instructions emphasized accuracy, and so encouraged participants to wait for evidence accumulated via feedback.

We interpret these findings as showing a selective need for increased detailed visual analysis of high complexity scenes. For simple scenes, the initial, coarse representation provided by the feed-forward sweep is enough to obtain information about the presence of a target object. For complex scenes, however, the feed-forward sweep is not sufficiently informative: it merely signals that there is a lot of ‘stuff’ in the scene, and detecting the target object requires additional processing.

### Global-to-local processing and attention

This interpretation is consistent with the global-to-local processing frameworks which suggest that detailed scene analysis takes place via reentrant processing [23,50–52]. These frameworks propose that the feed-forward sweep provides visual cortex with a *base representation*. If the visual task can be solved based on this representation alone, no further operations are necessary and action selection can be initiated. If it is not sufficiently informative, *elemental operations* are applied such as contour grouping and texture segmentation, which integrate individual line segments and other features encoded in low-level areas via incremental grouping [21,22]. We believe that the differential neural response observed in our fMRI and EEG data for targets relative to non-target scenes reflects the implementation of these elemental operations. A psychological correlate of this feedback-driven process could be termed ‘attentive processing’ while the feed-forward stage could be considered pre-attentive [53]; see [21] for a detailed discussion. Our observation of an increased fMRI response to targets is clearly consistent with numerous observations that attentional modulations are reflected in response enhancements in visual cortex [54]. Importantly, however, our results suggest that this response enhancement is *selectively applied* on a trial-by-trial basis to scenes which have high complexity, as determined by scene statistics.

### The impact of recurrent processing on behavior

Our behavioral observations support the presence of selectively increased feedback for complex scenes in two ways. First, in Experiment 1 we observed that scene complexity influenced response selection, but not response suppression [55–58]. Specifically, we observed that the decision time was prolonged for complex scenes, suggesting a slower rate of information accumulation. In the Experiment 2 we examined this hypothesis through the manipulation of time restrictions. Behaviorally, we showed that while RTs were always increased for highly complex scenes, accuracy only declined when the instructions emphasized a speeded response. The drift diffusion model [43,44,59] was then used to show that drift rate (the latent variable capturing the rate of information accumulation) was indeed slower for highly complex scenes [60–62]. In addition, we observed that drift rate was related to single-trial feedback-driven ERP responses for high, but not low or medium complexity scenes. This reliance on feedback provides an explanation for the difference in behavioral performance when participants emphasized speed above accuracy (and thus were not able to incorporate the information provided by the feedback response). Moreover, this ERP-drift rate relation is consistent with reports of a ‘discriminating component’ around 300 ms reflecting the amount of sensory evidence for perceptual decisions on objects in phase noise [61,63]. Taken together, these observations underline the importance of feedback activity for processing complex scenes to optimize behavioral performance.

### A role for scene statistics in signaling the need for recurrent processing

We propose that the complexity of the visual input can be estimated using image statistics derived from contrast distributions, in particular contrast energy and spatial coherence. These statistics are potentially suitable computational substrates for such a representation because they can be computed directly from local contrast responses in e.g. LGN [35]. These parameters strongly affect the amplitude of evoked EEG activity early in time in visual processing [35,37,64] suggesting they are indeed available at early stages of visual computation and could therefore serve as ‘markers’ to determine whether further visual operations are necessary. This idea is reminiscent of previous proposals suggesting that scenes are summarized as coarse ‘blobs’ that precede detailed analysis at smaller spatial scales depending on their diagnostic value [65,66], or via low spatial frequencies that are used to direct top-down facilitation [67]. It is also consistent with results showing that feedback is necessary to model categorization of degraded scenes [26] and with results reported by [30], who found that masking effects were stronger for scenes that were ‘less easily segmented’.

Here, rather than filtering, degrading or otherwise manipulating the scenes, we used scene statistics to sample variation in scene complexity, providing a quantitative computational approach to estimate this property. We sampled scenes with low, intermediate or high CE and SC values, assuming a linear relation between these values and scene complexity. We note, however, that several of our results, in particular the fMRI responses in higher-order regions and behavioral accuracy in Experiment 1, appeared to show a U-shaped, rather than linear modulation across conditions, suggesting that CE and SC are not a straightforward parametric index of scene complexity. In particular, while the high condition consistently deviated from the medium condition in terms of both behavioral (higher RT, lower accuracy) and neural (increased differential V1 fMRI and late ERP responses) effects, the low condition sometimes also showed a decrease in performance, but without a clear neural correlate. Given the clear neural effects for the high condition, the main focus of this paper is on that condition. However, a separate investigation of the U-shaped effects based on an additional set of behavioral studies [68] shows that behavioral performance is indeed often optimal for scenes with intermediate, rather than low, scene complexity. One potential explanation for this benefit is that feed-forward processing for intermediate CE/SC scenes is faster or more efficient due to, for example, increased familiarity with such scenes. However, intermediate scenes could also contain more or better contextual cues regarding the presence of target objects, resulting in increased interaction between scene and object processing pathways [69–71]. In sum, our results do not provide a clear explanation for this pattern, and future research is needed to determine the neural underpinnings of the improved performance for intermediate relative to low complexity images as defined by the CE/SC parameterization.

### How does the visual system determine whether recurrent processing is needed?

A question that remains unresolved in this study is which, if any, brain areas might be involved in computing scene complexity, i.e. which region ‘reads out’ the scene statistics from the population response in early visual regions. Consistent with previous studies [72,73], we found a strong distinction between animal- and non-animal images in object-selective LOC and face-selective FFA. However, this differential activity was not strongly modulated by whether the object was embedded in a complex scene or a simple scene. We did observe, however, an effect of scene complexity in PPA. While the scene-selectivity of PPA is commonly attributed to coding of 3D spatial layout [74–76] it is also sensitive to object information [77–79], as well as low-level features such as spatial frequency, contrast, rectilinearity and texture [79–84], suggesting that PPA may be a suitable region to demonstrate an influence of the broader scene context on object-related activity [69]. Importantly, PPA is biased towards the visual periphery [85,86], containing relatively large receptive fields [87,88] making it suited for computation of larger-scale summary statistics of the input [89]. Consistently, a recent study found that PPA was sensitive to difference in scene complexity as defined by various image-computable computational measures such as image compression and self-similarity [90].

Based on this prior literature, we speculate that the enhanced recurrent processing in early visual regions is initiated based on a feed-forward, summary statistics based computation of scene complexity in PPA. However, future research using for example time-resolved measurements of PPA activity will be necessary to confirm or deny the presence of such a representation in PPA during object detection in natural scenes.

### Influence of task on target detection in complex scenes

Perceiving real-world scenes involves more than detection of objects: for example, we can recognize a scene as a specific place, or determine its navigability [91]. It is unclear whether feedback is ‘intrinsically’ enhanced upon perceiving a scene of high complexity regardless of observer goal, or whether it is selectively enhanced when a participant is actively searching for a target object in a complex scene. One way to test this is to compare the current results to a situation in which participants see scenes of varying levels of complexity while performing a task without any object detection requirement (e.g. an orthogonal visual task at fixation, or detecting a concurrent auditory signal). Such top-down task manipulations have been shown to affect ERP responses to natural scenes in recurrent processing time windows [64], and feedback is thought to be involved in the application of attentional templates [92,93], which may differ between different tasks [94] or the level of detail necessary to solve the task [66] and therefore in the amount of feedback required. While we varied scene complexity on a trial-by-trial basis, making it difficult for participants to use a top-down strategy to ‘predict’ how much attention they would need to direct to solve that trial, they still needed to apply a search target in order to solve the task. This search target was the same throughout the entire experiment (animal), but our whole-brain fMRI analysis (Fig 3) did indicate some target-related activity differences for high complexity scenes in posterior parietal regions, which have been associated both with representing attentional templates as well as outcomes of attentional selection [92]. A deeper understanding of the interaction between scene complexity and top-down task requirements and their respective representation in cortical regions requires future experimental study. So far, however, our results suggest that although object recognition based on feed-forward information [5] may be possible for simple stimuli, detection of objects in complex real-world scenes additionally involves feedback processing.

## Materials and methods

### Experiment 1 (fMRI)

#### Ethics statement

All participants provided written informed consent and were financially compensated. The experiment was approved by the ethics committee of the University of Amsterdam.

#### Subjects

Twenty-five participants (7 males, age 19–26 years; mean = 21.6, SD = 1.7, normal or corrected-to-normal vision) took part in the fMRI experiment. One participant had a median reaction time of 2 standard deviations above average as well as 7.5% non-responses and was therefore excluded from further data analysis. Another participant was excluded because of excessive head movement (absolute displacement of twice the voxel size caused by multiple movements across a single experimental run).

#### Stimuli

Scenes were selected from a larger set of 4800 scenes used in a previous EEG study [95]. The larger dataset contained scenes from several online databases, including the INRIA holiday database [96], the GRAZ dataset [97], ImageNet [98], and the McGill Calibrated Color Image Database [99]. For each scene, one CE and one SC value was computed by simulating the output of contrast-sensitive receptive fields and integrating these responses across the scene by averaging (CE) and divisive normalization (SC). In natural scenes, CE and SC correlate strongly with parameters of a Weibull function fitted to the distribution of contrast values [35,36], which is informative about the degree of scene fragmentation [34]. CE is an approximation of the distribution width (the scale parameter of the function), informing about the overall presence of edges in an image, whereas SC is an approximation of its shape (the degree to which the function describes a power law or a Gaussian distribution), capturing higher-order correlations between edges. As a result, images with low CE/SC values have strong ‘Inherent’ figure-ground segmentation, often containing a large (central) object surrounded by a homogenous background (and therefore containing few edges that are spatially correlated because they belong to the same object), whereas images with high CE/SC values are more complex, and typically textured or cluttered (with many edges distributed in a Gaussian manner that are uncorrelated); while they can contain large objects, they tend to have a background consisting of uncorrelated edge ‘noise’ (see also De Cesarei et al., (2017), footnote 4). The model is described in more detail in [37,64].

Here, we used these image statistics to selectively sample scenes with various levels of complexity. We created three conditions: LOW, MEDIUM and HIGH (Fig 1*B*), whereby each condition was defined by its CE/SC values. For the LOW and HIGH condition, we selected the lower and upper 25% percentiles of the distribution of CE/SC values in the full stimulus set, respectively; for the MEDIUM condition, the middle 35% percentile. Each condition consisted of 160 images, half of which contained an animal. Importantly, target and non-target images were matched *within condition* in their CE and SC values such that animal and non-animal images did not differ from each other in their mean (all t(158) < 0.13, all p > 0.89) or median values (Wilcoxon rank sum test all z < 0.16, all p > 0.87). Images were randomly selected from the larger set of scenes solely based on their image statistics and annotations (animal/non-animal). Animal images contained a wide variety of animals including pets such as dogs and cats but also wildlife, reptiles and fish. Non-animal images consisted of urban, landscape and indoor scenes and contained a variety of objects, ranging from vehicles to household items. Several representative exemplars from each condition are provided in Fig 1*C*.

#### Experimental design

Participants performed the animal vs. non-animal categorization task in a stop signal paradigm (Fig 1*D*). Our motivation for using this paradigm was based on a separate research question inspired by a previous set of studies [56,100], which asked whether the quality of visual input affects decision-making and response inhibition. However, analysis of the behavioral data showed scene complexity to only modulate decision-making on trials without stop-signals [55–58]. Given that the focus of the current study is on detection performance rather than response inhibition, trials with a stop-signal presentation were excluded from analysis. Each participant performed 480 trials in total, divided over 2 separate runs. Each trial lasted 2000 ms and started with a fixation cross of variable duration (500–750 ms jittered with 50 ms intervals), after which a scene (640x480 pixels) was presented for 100 ms. The scene stimuli were back-projected on a 61x36 cm LCD screen that was viewed through a mirror attached to the head coil at ~120 cm viewing distance using Presentation software (Neurobehavioral Systems, Albany, CA, USA). There were two trial types: GO and STOP trials. On GO trials, participants had to indicate whether the stimulus was an animal or non-animal scene before the trial ended (i.e. at maximum within 1250 ms) by pressing one of two buttons. They indicated their response using a hand-held button box with the index or middle finger. If they did not respond in time, a screen displaying the word ‘miss’ appeared for 2000 ms. On STOP trials a beep signal was presented over the headphones indicating that the participant had to withhold their response. At the start of the experiment, the time interval between the stimulus and beep (stop signal delay) was initialized at 250 ms and was adjusted in a staircase procedure based on the stopping performance [101]). Trials were presented in two randomized sequences that were counterbalanced across participants. Overall, 25% of the scenes (60 animal, 60 non-animal) were shown in STOP trials. The same set of scenes was used in the STOP trials for all participants, and these scenes were excluded from analysis. Thus, all analyses reported here included only the GO trials. For these trials, animal and non-animal scenes were still matched in their CE and SC values per condition (means: all t(118) < 1.13, all p > 0.26; medians: all z < 1.10, all p > 0.28).

#### fMRI data acquisition

BOLD-MRI data was acquired in a single scanning session, over the course of two runs on a Philips Achieva XT 3T scanner with a 32-channel head-coil located at the University of Amsterdam, The Netherlands. In each run 255 T2*-weighted GE-EPI recordings (TR = 2000 ms, TE = 27.6 ms, FA = 76.1°, SENSE = 2, FOV = 240 mm^2^, matrix size = 80^2^, 37 slices, slice thickness 3 mm, slice gap = 0.3 mm) were made. Breathing rate and heartbeat (using the pulse-oxidization signal recorded from the tip of one of the participant’s fingers) was measured during fMRI acquisition. In addition, a separate functional localizer scan was recorded (317 T2*weighted echo-planar images; TR = 1500 ms, TE = 27.6, FA = 70°, FOV = 240*79.5*240, matrix size = 96^2^, 29 slices, slice thickness 2.5mm, slice gap = 0.25) in which participants viewed a series of houses, faces, objects as well as phase-scrambled scenes while pressing a button when an image was directly repeated (12.5% likelihood). These separate localizer scans were used to identify the following regions-of-interest (ROIs): the fusiform face area (FFA), the parahippocampal place area (PPA) and lateral occipital complex (LOC). Finally, a 3D-T1 weighted scan (TE = 3.8 ms, TR = 8.2 ms, FA = 8°, FOV = 256^2^, matrix size = 256^2^, 160 sagittal slices, slice thickness = 1mm) was acquired after the functional runs. This scan was used to register the functional volumes of each run to the structural brain, after which they were registered to standard MNI (Montreal Neurological Institute) space.

#### fMRI analysis: Animal/non-animal categorization

Analysis was performed using FEAT (FMRI Expert Analysis Tool) Version 6.00, part of FSL (FMRIB’s Software LIbrary, www.fmrib.ox.ac.uk/fsl) and custom Matlab code. The functional data were motion- [102] and slice-time corrected. A temporal median filter was applied to remove low frequencies, after which the data was spatially smoothed with Gaussian kernel at a 5 mm FWHM. The preprocessed scans were subjected to voxel-wise event-related GLM analysis using FILM [103] by convolving the onset times of each trial with a double gamma function to model the hemodynamic response function. We generated explanatory variables (EVs) according to the following conditions: LOW animal, LOW non-animal, MEDIUM animal, MEDIUM non-animal, HIGH animal, and HIGH non-animal. For these EVs, only correct GO trials were included; STOP, omission, and error trials were modeled as separate EVs. In addition, heartbeat and breathing measurements were included in the GLM as nuisance variables. This resulted in an estimate of the BOLD signal for each EV in each voxel, based on which the following contrasts of interest were computed: LOW (animal > non-animal), MED (animal > non-animal) and HIGH (animal > non-animal). As the trial design contained insufficient spacing between trials to estimate a baseline condition, all analyses were conducted on these differential activity measures.

#### fMRI analysis: Localizer scans

The localizer scans were preprocessed for the purpose of another study conducted within the same experimental session [56]. Again, the data were motion- and slicetime corrected and prewhitened. In addition, they were spatially smoothed using a 4mm Gaussian filter and were temporally filtered by means of a high-pass filter (sigma = 50s). A GLM was fitted with following EVs: for FFA, faces > (houses and objects), for PPA, houses > (faces and objects) and for LOC, intact images > scrambled images. The resulting statistical maps were masked with anatomically-defined regions from the Harvard-Oxford cortical-structural atlas implemented in FSL. For FFA, these were the temporal occipital and occipital fusiform gyrus; for PPA, the parahippocampal gyrus and lingual gyrus, allowing activity to extend posteriorly up to MNI coordinate y = -74; for LOC, the lateral occipital cortex inferior division. Significant voxels within these masks were thresholded at z = 2.3 (FFA and PPA) or z = 3.0 (LOC). For one participant, neither left nor right LOC could be reliably identified: the ROI results for LOC are thus based on 22 instead of 23 participants.

#### Statistical analysis: Behavioral data

Mean RT and accuracy (percentage correct) was computed for each participant based on the GO trials. Differences in accuracy and RT between the three conditions (LOW, MEDIUM, HIGH) were statistically evaluated using repeated-measures ANOVAs. Significant main effects were followed up by two-tailed, post-hoc pairwise comparisons using a Sidák correction (a variant of Bonferroni correction; [104–106]) at α = 0.05, and the p-values reported in the main text are the Sidák adjusted p-values. Data were analyzed in Matlab (Mathworks, Natick, MA, USA) and SPSS 22.0 (IBM, Armonk, USA).

#### Statistical analysis: Whole brain

Whole brain maps were computed by contrasting animal and non-animal responses for each of the three conditions (LOW, MEDIUM, and HIGH). The resulting maps were first pooled across runs (fixed effects) and then across subjects (mixed effects using FLAME1 [107];. after which the following contrasts were run: HIGH (animal > non-animal) > LOW (animal > non-animal); HIGH (animal > non-animal) > MEDIUM (animal > non-animal); and MEDIUM (animal > non-animal) > LOW (animal > non-animal). Results were corrected for multiple comparisons using cluster correction implemented in FSL (z = 2.3, p < 0.05; [108]).

#### Statistical analysis: ROIs

We additionally examined the contrasts of interest within *a priori* defined regions-of-interest (ROIs) derived from the functional localizer scans (see above) as well as an anatomical mask of V1 derived from the Jülich histological atlas implemented in FSL [109]. Because initial inspection of the results did not indicate major differences between hemispheres, all results are reported for bilateral ROIs. T-values obtained for the contrast of interest were averaged over voxels within each ROI. The resulting averaged activity values were compared across conditions using one-way repeated-measures ANOVAs. Significant main effects were followed up by post-hoc pairwise comparisons between conditions with a Sidák correction at α = 0.05. Data were analyzed in Matlab (Mathworks, Natick, MA, USA) and SPSS 22.0 (IBM, Armonk, USA).

### Experiment 2 (EEG)

#### Subjects

Twenty-eight participants (8 males, 19–25 years old, mean = 21.9, SD = 1.9) took part in the EEG experiment. All participants had normal or corrected-to-normal vision, provided written informed consent and received financial compensation. The ethics committee of the University of Amsterdam approved the experiment. Two participants were excluded in preprocessing: one based on behavior (average RT 2 standard deviations from the group average) as well as bad EEG quality (excessive muscle tension reflected in the EEG signal as high frequency noise), and another due to a technical issue which led to duplicate markers being written into the EEG signal.

#### Experimental design

Participants viewed the 480 scene stimuli in randomized orders while performing an animal / non-animal speed-accuracy categorization task on the same images used for the fMRI experiment (Fig 1*E*). Stimuli were presented on a 19-inch ASUS monitor with a frame rate of 60 Hz and a screen resolution of 1920 x 1080 pixels. Participants were seated 90 cm from the monitor such that stimuli subtended ~14x10° of visual angle. On each trial, one image was randomly selected and presented in the center of the screen on a grey background for 100 ms. Between trials, a fixation-cross was presented with a semi-randomly chosen duration of either 350, 400, 450, 500 or 550 ms, averaging to 450 ms. Participants searched for animals under either speed or accuracy instructions in randomly alternating blocks that each consisted of 20 trials. Each mini block started with the presentation of an instruction screen displaying either the words ‘QUICK!' for speeded blocks, or ‘ACCURATE!’ (in Dutch) for accuracy blocks for a duration of 5000 ms. In addition, before every trial, the instruction appeared again for 100 ms. Every image was presented twice, once under a speed instruction, and once under an accuracy instruction. After every 120 trials, participants took a short break. To exclude effects of response preparation, keyboard buttons were switched half-way in the experiment. Choices and RTs with respect to the start of the presentation of the image were recorded. For both instruction types, participants received feedback on their performance. On the speed trials, participants were presented with “too slow” feedback in case they failed to respond in time (<500 ms), and “on time” when they were quick enough. On the accuracy trials, participants were presented with “correct” and “incorrect” feedback. Stimuli were presented using Presentation software (version 17.0, Neurobehavioral Systems, Inc).

#### EEG data acquisition

EEG was recorded with a 64-channel Active Two EEG system (Biosemi Instrumentation, Amsterdam, The Netherlands, www.biosemi.com) at a sample rate of 2048 Hz. The EEG setup was similar to that of our previous studies [37,39,64]. In short, we used caps with an extended 10–20 layout modified with 2 additional occipital electrodes (I1 and I2, which replaced F5 and F6). Eye movements were recorded with additional electrooculograms (EOG). Preprocessing was done in Brain Vision Analyzer 2 (BVA2) and included the following steps: 1) offline referencing to the average of two external electrodes placed on the earlobes; 2) a high-pass filter at 0.1 Hz (12 dB/octave), a low-pass filter at 30 Hz (24 dB/octave), and a notch filter at 50 Hz; 3) automatic removal of deflections larger than 250 mV (however, after visual inspection of the removed data segments, this threshold was raised for some participants for which data had erroneously been marked for removal due to the presence of high amplitude blinks, which were corrected for at step 5 below); 4) down sampling to 256 Hz; 5) ocular correction using semi-automatic independent component analysis (ICA) followed by visual inspection to identify the components related to eye blinks; 6) segmentation into epochs from -100 to 500 ms from stimulus onset; 7) baseline correction between -200 and 0 ms; 8) automated artifact rejection (maximal voltage 50 μV, minimal/maximal amplitudes -75/75 μV, lowest activity 0.50 μV); 9), conversion of the obtained ERPs to current source density responses [110]. For the temporal filtering, phase shift-free Butterworth filters implemented in BVA2 were used. Median rejection rate across subjects was 94.5 out of 960 ERP trials (min 8 trials, max 486 trials); in total, 13.6% of the data was rejected. After preprocessing, the ERPs were imported into MatLab (Mathworks, Natick, MA, USA) for statistical analysis.

#### Statistical analysis: Behavioral data

Choice accuracy and reaction times (RTs) were computed separately for the speed and accurate blocks. Fast guesses (RTs < 150 ms) or RTs > 3 standard deviations from the mean were removed before analysis (mean = 2.2%, SD = 0.9%, min = 0.8%, max = 4.2%). Differences between the LOW, MEDIUM and HIGH condition were tested using two-factor repeated-measures ANOVAs. Significant main effects were followed up by post-hoc pairwise comparisons between conditions or tasks using Sidák multiple comparisons correction at α = 0.05. Data were analyzed in Matlab (Mathworks, Natick, MA, USA) and SPSS 22.0 (IBM, Armonk, USA).

#### Drift diffusion modeling

Based on go trial RT distributions of both correct responses and errors, the formal Ratcliff drift diffusion model (DDM) estimates the speed of evidence accumulation, ‘drift rate’, the variability of evidence accumulation ƞ, the amount of evidence needed for a decision (a), the starting point of evidence accumulation (z), and the variability of this starting point (S_z_). Together these parameters generate a distribution of decision times (DT). However, observed reaction times (RT) are also thought to contain non-stimulus specific components such as response preparation and motor execution, which combine in the parameters non-decision time (T_er_), and non-decision time variability (S_t_). In general, DDM assumes that (T_er_) simply shifts the distribution of DT such that: RT = DT+ T_er_ [44,111,112].

To analyze the RT data of the EEG experiment with the drift diffusion model we used a recently developed hierarchical Bayesian estimation of DDM parameters (HDDM) implemented in Python (version 0.6.0, see http://ski.clps.brown.edu/hddm_docs/), which allows the simultaneous estimation of subject and group parameters and thus requires less data per subject [45]. To gain a deeper insight into how scene complexity affects choice RT, we investigated a model where the speed of information accumulation (v) and the amount of evidence required to reach a choice (a) were both allowed to vary across the three natural scene conditions (LOW, MED, HIGH). Moreover, because previous work has consistently shown that participants require more evidence to reach a choice during accurate instruction trials (as compared to speed trials), evidence requirements (a) was additionally allowed to vary across ‘speed’ or ‘accurate’ instruction trials [113,114]. For this model, three chains of 20,000 samples were generated from the posteriors. In order to assure chain convergence, the first 5,000 samples were discarded (burn), resulting in a trace of 15000 samples for each chain. These chains were then tested for convergence using the Gelman-Rubin statistic, which compares the intra-chain variance of the model to the intra-chain variance of different runs of the same model. All chains were converged and all Rhats were close to 1 [115].

#### Statistical analysis: EEG

For each individual subject we computed the average ERP to animal and non-animal scenes in each of the three conditions. Following previous EEG studies on figure-ground segmentation [18,46], ERPs were pooled across a set of occipital and peri-occipital electrodes overlying visual cortex (O1, O2, Oz, POz, PO3, PO4, PO6, PO7, PO8). Per condition, difference waves between animal and non-animal scenes were tested against zero using two-tailed, one-sample t-tests. Difference waves were compared across conditions using the following two-tailed, paired-samples t-tests: HIGH (animal > non-animal) vs. LOW (animal > non-animal); HIGH (animal > non-animal) vs. MEDIUM (animal > non-animal); and MEDIUM (animal > non-animal) vs. LOW (animal > non-animal). Given the large number of statistical comparisons, the results were corrected for multiple comparisons across the two task-instructions, conditions and time-points by means of FDR correction at α = 0.01 using an empirically derived FDR-corrected threshold of *q* = 0.0014).

To assess the trial-by-trial linear relationship between the EEG amplitudes and drift rate, we fitted a HDDM regression model [45] to the RT distributions for target and non-target decisions (referred to as ‘response-coded’ in HDDM), across both speeded and accurate trials, with LOW, MED and HIGH coded as categorical variables. As the ERP predictor per trial, we took the average ERP amplitude between the first significant deflection until the peak amplitude of the average HIGH difference wave for speed and accurate trials (220–325 ms). We ran four separate chains with 20,000 samples. The first 7500 samples were discarded (burn) and every 10th sample (thin) was concatenated, resulting in a trace of 5000 samples. The model was tested for convergence using the Gelman-Rubin statistic, which compares the intra-chain variance of the model to the intra-chain variance of the different runs. All chains were converged and all values were close to 1. Since HDDM uses Bayesian estimation to obtain the posterior probability densities of the regression weights, the p-values for the distributions in Fig 7 were analyzed directly for hypothesis testing. Specifically, the p-value for the significance of each regression weight reflects the percentage of the posterior probability distribution that crosses the zero point. The p-value for the test between distribution reflects the percentage of distribution 1 (e.g., LOW) that crosses the mean of distribution 2 (e.g., MED), see http://ski.clps.brown.edu/hddm_docs/howto.html#hypothesis-testing.

## Supporting information

S1 Fig Influence of non-animal image content on behavioral and EEG results from Experiment 2. A) Behavioral reaction time and accuracy as a function of complexity (LOW, MED, HIGH) and task instruction (speeded or accurate) when only including (left) or excluding (right) non-animal scenes with vehicles, humans and man-made objects (manually annotated). B-C) Differences in ERP amplitude for animal and non-animal scenes for LOW, MED, and HIGH complexity scenes, computed with (left) or without (right) scenes with vehicles, humans and man-made objects, separately for speed (B) and accurate (C) instructions. The ‘with vehicles, humans, objects’ analysis included 51 non-animal trials for LOW, 58 scenes for MED, and 57 scenes for HIGH; the ‘without vehicles, humans, objects’ analysis included 29 trials for LOW, 22 trials for MED, and 23 trials for HIGH.
(TIF)

## References

[1] VanRullen R, Thorpe SJ. Surfing a spike wave down the ventral stream. Vision Res. 2002;42: 2593–615. 12446033 10.1016/s0042-6989(02)00298-5

[2] Liu H, Agam Y, Madsen JR, Kreiman G. Timing, timing, timing: fast decoding of object information from intracranial field potentials in human visual cortex. Neuron. 2009;62: 281–90. 10.1016/j.neuron.2009.02.025 19409272 PMC2921507

[3] Thorpe S, Fize D, Marlot C. Speed of processing in the human visual system. Nature. 1996;381: 520–522. 10.1038/381520a0 8632824

[4] VanRullen R, Thorpe S. The time course of visual processing: from early perception to decision-making. J Cogn Neurosci. 2001;13: 454–61. 11388919 10.1162/08989290152001880

[5] Serre T, Oliva A, Poggio T. A feedforward architecture accounts for rapid categorization. Proc Natl Acad Sci. 2007;104: 6424–9. 10.1073/pnas.0700622104 17404214 PMC1847457

[6] Poggio T, Serre T. Models of visual cortex. Scholarpedia. 2013;8: 3516.

[7] Yamins DLK, Hong H, Cadieu C, Dicarlo JJ. Hierarchical Modular Optimization of Convolutional Networks Achieves Representations Similar to Macaque IT and Human Ventral Stream. Adv Neural Inf Process Syst. 2013;26: 3093–3101.

[8] Khaligh-Razavi SM, Kriegeskorte N. Deep Supervised, but Not Unsupervised, Models May Explain IT Cortical Representation. PLoS Comput Biol. 2014;10 10.1371/journal.pcbi.1003915 25375136 PMC4222664

[9] Güçlü U, van Gerven MAJ. Deep Neural Networks Reveal a Gradient in the Complexity of Neural Representations across the Ventral Stream. J Neurosci. 2015;35: 10005–10014. 10.1523/JNEUROSCI.5023-14.2015 26157000 PMC6605414

[10] Rockland KS, Pandya DN. Laminar origins and terminations of cortical connections of the occipital lobe in the rhesus monkey. Brain Res. 1979;179: 3–20. 116716 10.1016/0006-8993(79)90485-2

[11] Salin PA, Bullier J. Corticocortical connections in the visual system: structure and function. Physiol Rev. 1995;75: 107–54. 10.1152/physrev.1995.75.1.107 7831395

[12] Van Essen DC, Maunsell JHR. Hierarchical organization and functional streams in the visual cortex. Trends Neurosci. 1983;6: 370–375. 10.1016/0166-2236(83)90167-4

[13] Kravitz DJ, Saleem KS, Baker CI, Ungerleider LG, Mishkin M. The ventral visual pathway: an expanded neural framework for the processing of object quality. Trends Cogn Sci. Elsevier Ltd; 2013;17: 26–49. 10.1016/j.tics.2012.10.011 23265839 PMC3532569

[14] Lamme VAF. The neurophysiology of figure-ground segregation in primary visual cortex. J Neurosci. 1995;15: 1605–1615. 7869121 10.1523/JNEUROSCI.15-02-01605.1995 PMC6577835

[15] Zipser K, Lamme VAF, Schiller PH. Contextual modulation in primary visual cortex. J Neurosci. 1996;16: 7376–89. 8929444 10.1523/JNEUROSCI.16-22-07376.1996 PMC6578953

[16] Self MW, van Kerkoerle T, Supèr H, Roelfsema PR. Distinct Roles of the Cortical Layers of Area V1 in Figure-Ground Segregation. Curr Biol. 2013;23: 2121–2129. 10.1016/j.cub.2013.09.013 24139742

[17] Poort J, Self MW, van Vugt B, Malkki H, Roelfsema PR. Texture Segregation Causes Early Figure Enhancement and Later Ground Suppression in Areas V1 and V4 of Visual Cortex. Cereb cortex. 2016;26: 3964–3976. 10.1093/cercor/bhw235 27522074 PMC5028009

[18] Wokke ME, Sligte IG, Steven Scholte H, Lamme VAF. Two critical periods in early visual cortex during figure-ground segregation. Brain Behav. 2012;2: 763–777. 10.1002/brb3.91 23170239 PMC3500463

[19] Heinen K, Jolij J, Lamme VAF. Figure-ground segregation requires two distinct periods of activity in V1: a transcranial magnetic stimulation study. Neuroreport. 2005;16: 1483–1487. 10.1097/01.wnr.0000246328.96932.4c 16110276

[20] Wokke ME, Vandenbroucke ARE, Scholte HS, Lamme VAF. Confuse your illusion: feedback to early visual cortex contributes to perceptual completion. Psychol Sci. 2013;24: 63–71. 10.1177/0956797612449175 23228938

[21] Roelfsema PR. Cortical algorithms for perceptual grouping. Annu Rev Neurosci. 2006;29: 203–27. 10.1146/annurev.neuro.29.051605.112939 16776584

[22] Roelfsema PR, Lamme VAF, Spekreijse H. The implementation of visual routines. Vision Res. 2000;40: 1385–411. 10788648 10.1016/s0042-6989(00)00004-3

[23] Lamme V, Roelfsema P. The distinct modes of vision offered by feedforward and recurrent processing. Trends Neurosci. 2000;23: 571–9. 11074267 10.1016/s0166-2236(00)01657-x

[24] Koivisto M, Railo H, Revonsuo A, Vanni S, Salminen-Vaparanta N. Recurrent processing in V1/V2 contributes to categorization of natural scenes. J Neurosci. 2011;31: 2488–92. 10.1523/JNEUROSCI.3074-10.2011 21325516 PMC6623680

[25] Camprodon JA, Zohary E, Brodbeck V, Pascual-Leone A. Two phases of V1 activity for visual recognition of natural images. J Cogn Neurosci. 2010;22: 1262–9. 10.1162/jocn.2009.21253 19413482 PMC3369215

[26] Wyatte D, Curran T, O’Reilly R. The limits of feedforward vision: recurrent processing promotes robust object recognition when objects are degraded. J Cogn Neurosci. 2012;24: 2248–61. 10.1162/jocn_a_00282 22905822

[27] Lamme VAF, Zipser K, Spekreijse H. Masking interrupts figure-ground signals in V1. J Cogn Neurosci. 2002;14: 1044–53. 10.1162/089892902320474490 12419127

[28] Fahrenfort JJ, Scholte HS, Lamme VAF. Masking disrupts reentrant processing in human visual cortex. J Cogn Neurosci. 2007;19: 1488–97. 10.1162/jocn.2007.19.9.1488 17714010

[29] van Loon AM, Scholte HS, Van Gaal S, van der Hoort BJJ, Lamme VAF. GABA A agonist reduces visual awareness: A masking—EEG experiment. J Cogn Neurosci. 2012;24: 965–974. 10.1162/jocn_a_00197 22264199

[30] Koivisto M, Kastrati G, Revonsuo A. Recurrent processing enhances visual awareness but is not necessary for fast categorization of natural scenes. J Cogn Neurosci. 2013;26: 223–231. 10.1162/jocn_a_00486 24047378

[31] De Cesarei A, Loftus GR, Mastria S, Codispoti M. Understanding natural scenes: Contributions of image statistics. Neurosci Biobehav Rev. Elsevier Ltd; 2017;74: 44–57. 10.1016/j.neubiorev.2017.01.012 28089884

[32] Torralba A, Oliva A. Statistics of natural image categories. Netw Comput Neural Syst. 2003;14: 391–412. 10.1088/0954-898X/14/3/302 12938764

[33] Oliva A. Gist of the scene. Neurobiology of Attention. 2005 pp. 251–257.

[34] Geusebroek J-M, Smeulders AWM. Fragmentation in the vision of scenes. Proc Ninth IEEE Int Conf Comput Vis. Ieee; 2003; 130–135 vol.1 10.1109/ICCV.2003.1238326

[35] Scholte HS, Ghebreab S, Waldorp L, Smeulders AWM, Lamme VAF. Brain responses strongly correlate with Weibull image statistics when processing natural images. J Vis. 2009;9: 1–15. 10.1167/9.4.29 19757938

[36] Ghebreab S, Smeulders AWM, Scholte HS, Lamme VAF. A biologically plausible model for rapid natural image identification. Advances in Neural Information Processing Systems. 2009 pp. 629–637.

[37] Groen IIA, Ghebreab S, Prins H, Lamme VAF, Steven Scholte H, Scholte HS. From image statistics to scene gist: evoked neural activity reveals transition from low-level natural image structure to scene category. J Neurosci. 2013;33: 18814–24. 10.1523/JNEUROSCI.3128-13.2013 24285888 PMC6618700

[38] Groen IIA, Ghebreab S, Lamme VAF, Scholte HS. Low-level contrast statistics are diagnostic of invariance of natural textures. Front Comput Neurosci. 2012;6: 34 10.3389/fncom.2012.00034 22701419 PMC3370418

[39] Groen IIA, Ghebreab S, Lamme VAF, Scholte HS. Spatially pooled contrast responses predict neural and perceptual similarity of naturalistic image categories. PLoS Comput Biol. Public Library of Science; 2012;8: e1002726 10.1371/journal.pcbi.1002726 23093921 PMC3475684

[40] Harel A, Groen IIA, Kravitz DJ, Deouell LY, Baker CI. The time course of scene processing: A multi-faceted EEG investigation. eNeuro. 2016;3: e0139–16.2016. 10.1523/ENEURO.0139-16.2016 PMC5037322 27699208

[41] Ghodrati M, Ghodousi M, Yoonessi A. Low-Level Contrast Statistics of Natural Images Can Modulate the Frequency of Event-Related Potentials (ERP) in Humans. Front Hum Neurosci. 2016;10: 1–12. 28018197 10.3389/fnhum.2016.00630 PMC5145888

[42] De Cesarei A, Cavicchi S, Micucci A, Codispoti M. Categorization Goals Modulate the Use of Natural Scene Statistics. J Cogn Neurosci. 2018;xx: 1–17. 10.1162/jocn_a_01333 30188778

[43] Ratcliff R, Smith PL. Perceptual discrimination in static and dynamic noise: the temporal relation between perceptual encoding and decision making. J Exp Psychol Gen. 2010;139: 70–94. 10.1037/a0018128 20121313 PMC2854493

[44] Ratcliff R, McKoon G. The diffusion decision model: theory and data for two-choice decision tasks. Neural Comput. 2008;20: 873–922. 10.1162/neco.2008.12-06-420 18085991 PMC2474742

[45] Wiecki T V, Sofer I, Frank MJ. HDDM: Hierarchical Bayesian estimation of the Drift-Diffusion Model in Python. Front Neuroinform. 2013;7: 14 10.3389/fninf.2013.00014 23935581 PMC3731670

[46] Scholte HS, Jolij J, Fahrenfort JJ, Lamme VAF. Feedforward and recurrent processing in scene segmentation: electroencephalography and functional magnetic resonance imaging. J Cogn Neurosci. 2008;20: 2097–109. 10.1162/jocn.2008.20142 18416684

[47] Ratcliff R. A theory of memory retrieval. Psychol Rev. 1978;85: 59–108.

[48] Cavanagh JF, Wiecki T V., Cohen MX, Figueroa CM, Samanta J, Sherman SJ, et al Subthalamic nucleus stimulation reverses mediofrontal influence over decision threshold. Nat Neurosci. Nature Publishing Group; 2011;14: 1462–1467. 10.1038/nn.2925 21946325 PMC3394226

[49] Delis I, Dmochowski JP, Sajda P, Wang Q. Correlation of neural activity with behavioral kinematics reveals distinct sensory encoding and evidence accumulation processes during active tactile sensing. Neuroimage. 2018;175: 12–21. 10.1016/j.neuroimage.2018.03.035 29580968 PMC5960621

[50] Hochstein S, Ahissar M. View from the top: Hierarchies and reverse hierarchies in the visual system. Neuron. 2002;36: 791–804. 10.1016/S0896-6273(02)01091-7 12467584

[51] Petro LS, Vizioli L, Muckli L. Contributions of cortical feedback to sensory processing in primary visual cortex. Front Psychol. 2014;5: 1–8. 25414677 10.3389/fpsyg.2014.01223 PMC4222340

[52] Muckli L, De Martino F, Vizioli L, Petro LS, Smith FW, Ugurbil K, et al Contextual Feedback to Superficial Layers of V1. Curr Biol. The Authors; 2015;25: 2690–2695. 10.1016/j.cub.2015.08.057 26441356 PMC4612466

[53] Treisman AM, Gelade G. A feature-integration theory of attention. Cogn Psychol. 1980;136: 97–136. 10.1016/0010-0285(80)90005-5 7351125

[54] Kastner S, Ungerleider LG. Mechanisms of visual attention in the human cortex. Annu Rev Neurosci. 2000;23: 315–341. 10.1146/annurev.neuro.23.1.315 10845067

[55] Logan GD, Burkell J. Dependence and independence in responding to double stimulation: A comparison of stop, change, and dual-task paradigms. J Exp Psychol Hum Percept Perform. 1986;12: 549–563. 10.1037/0096-1523.12.4.549

[56] Jahfari S, Waldorp L, Ridderinkhof KR, Scholte HS. Visual information shapes the dynamics of corticobasal ganglia pathways during response selection and inhibition. J Cogn Neurosci. 2015;27: 1344–1359. 10.1162/jocn_a_00792 25647338

[57] Verbruggen F, Logan GD. Proactive adjustments of response strategies in the stop-signal paradigm. J Exp Psychol Hum Percept Perform. 2009;35: 835–854. 10.1037/a0012726 19485695 PMC2690716

[58] Jahfari S, Verbruggen F, Frank MJ, Waldorp LJ, Colzato L, Ridderinkhof KR, et al How Preparation Changes the Need for Top-Down Control of the Basal Ganglia When Inhibiting Premature Actions. J Neurosci. 2012;32: 10870–10878. 10.1523/JNEUROSCI.0902-12.2012 22875921 PMC6621019

[59] Wiecki T V, Frank MJ. A computational model of inhibitory control in frontal cortex and basal ganglia. Psychol Rev. 2013;120: 329–355. 10.1037/a0031542 23586447

[60] Sajda P, Philiastides MG, Heekeren HR, Ratcliff R. Linking Neuronal Variability to Perceptual Decision Making via Neuroimaging In: Ding D, Glanzman D, editors. The Dynamic Brain: an Exploration of Neuronal Variability and Its Functional Significance. Oxford scholarship online; 2011 pp. 214–231.

[61] Philiastides MG, Heekeren HR, Sajda P. Human Scalp Potentials Reflect a Mixture of Decision-Related Signals during Perceptual Choices. J Neurosci. 2014;34: 16877–16889. 10.1523/JNEUROSCI.3012-14.2014 25505339 PMC4261107

[62] Heekeren HR, Marrett S, Ungerleider LG. The neural systems that mediate human perceptual decision making. Nat Rev Neurosci. 2008;9: 467–479. 10.1038/nrn2374 18464792

[63] Philiastides MG, Ratcliff R, Sajda P. Neural representation of task difficulty and decision making during perceptual categorization: a timing diagram. J Neurosci. 2006;26: 8965–75. 10.1523/JNEUROSCI.1655-06.2006 16943552 PMC6675324

[64] Groen IIA, Ghebreab S, Lamme VAF, Scholte HS. The time course of natural scene perception with reduced attention. J Neurophysiol. 2016;115: 931–946. 10.1152/jn.00896.2015 26609116

[65] Schyns PG, Oliva A. From blobs to boundary edges: Evidence for time- and spatial-scale-dependent scene recognition. Psychol Sci. 1994;5: 195–200. 10.1111/j.1467-9280.1994.tb00500.x

[66] Oliva A, Schyns PG. Coarse blobs or fine edges? Evidence that information diagnosticity changes the perception of complex visual stimuli. Cogn Psychol. 1997;34: 72–107. 10.1006/cogp.1997.0667 9325010

[67] Bar M, Kassam KS, Ghuman AS, Boshyan J, Schmid AM, Dale AM, et al Top-down facilitation of visual recognition. Proc Natl Acad Sci USA. 2006;103: 449–454. 10.1073/pnas.0507062103 16407167 PMC1326160

[68] Seijdel N, Jahfari S, Groen IIA, Scholte HS. Low-level image statistics in natural scenes influence perceptual decision-making. 2018; 1–15. 10.17605/OSF.IO/P3R8A PMC7324621 32601499

[69] Brandman T, Vincent Peelen M. Interaction between scene and object processing revealed by human fMRI and MEG decoding. J Neurosci. 2017; 0582–17. 10.1523/JNEUROSCI.0582-17.2017 28687603 PMC6596648

[70] Aminoff EM, Kveraga K, Bar M. The role of the parahippocampal cortex in cognition. Trends Cogn Sci. Elsevier Ltd; 2013;17: 379–90. 10.1016/j.tics.2013.06.009 23850264 PMC3786097

[71] Kveraga K, Ghuman AS, Kassam KS, Aminoff EA, Hamalainen MS, Chaumon M, et al Early onset of neural synchronization in the contextual associations network. Proc Natl Acad Sci. 2011;108: 3389–3394. 10.1073/pnas.1013760108 21300869 PMC3044398

[72] Kriegeskorte N, Mur M, Ruff DA, Kiani R, Bodurka J, Esteky H, et al Matching categorical object representations in inferior temporal cortex of man and monkey. Neuron. Elsevier Ltd; 2008;60: 1126–41. 10.1016/j.neuron.2008.10.043 19109916 PMC3143574

[73] Naselaris T, Stansbury DE, Gallant JL. Cortical representation of animate and inanimate objects in complex natural scenes. J Physiol Paris. Elsevier Ltd; 2012;106: 239–49. 10.1016/j.jphysparis.2012.02.001 22472178 PMC3407302

[74] Epstein R, Kanwisher N. A cortical representation of the local visual environment. Nature. 1998;392: 598–601. 10.1038/33402 9560155

[75] Kravitz DJ, Peng CS, Baker CI. Real-world scene representations in high-level visual cortex: it’s the spaces more than the places. J Neurosci. 2011;31: 7322–7333. 10.1523/JNEUROSCI.4588-10.2011 21593316 PMC3115537

[76] Park S, Brady TF, Greene MR, Oliva A. Disentangling scene content from spatial boundary: complementary roles for the parahippocampal place area and lateral occipital complex in representing real-world scenes. J Neurosci. 2011;31: 1333–40. 10.1523/JNEUROSCI.3885-10.2011 21273418 PMC6623596

[77] Troiani V, Stigliani A, Smith ME, Epstein RA. Multiple object properties drive scene-selective regions. Cereb Cortex. 2014;24: 883–97. 10.1093/cercor/bhs364 23211209 PMC3948490

[78] Harel A, Kravitz DJ, Baker CI. Deconstructing visual scenes in cortex: gradients of object and spatial layout Information. Cereb Cortex. 2012;23: 947–957. 10.1093/cercor/bhs091 22473894 PMC3593580

[79] Cant JS, Xu Y. Object ensemble processing in human anterior-medial ventral visual cortex. J Neurosci. 2012;32: 7685–700. 10.1523/JNEUROSCI.3325-11.2012 22649247 PMC6703596

[80] Rajimehr R, Devaney KJ, Bilenko NY, Young JC, Tootell RBH. The “parahippocampal place area” responds preferentially to high spatial frequencies in humans and monkeys. PLoS Biol. 2011;9: e1000608 10.1371/journal.pbio.1000608 21483719 PMC3071373

[81] Nasr S, Tootell RBH. A cardinal orientation bias in scene-selective visual cortex. J Neurosci. 2012;32: 14921–6. 10.1523/JNEUROSCI.2036-12.2012 23100415 PMC3495613

[82] Watson DM, Hartley T, Andrews TJ. Patterns of response to visual scenes are linked to the low-level properties of the image. Neuroimage. Elsevier Inc.; 2014;99: 402–410. 10.1016/j.neuroimage.2014.05.045 24862072

[83] Kauffmann L, Ramanoël S, Guyader N, Chauvin A, Peyrin C. Spatial frequency processing in scene-selective cortical regions. Neuroimage. Elsevier Inc.; 2015; 10.1016/j.neuroimage.2015.02.058 25754068

[84] Lowe MX, Gallivan JP, Ferber S, Cant JS. Feature diagnosticity and task context shape activity in human scene-selective cortex. Neuroimage. Elsevier Inc.; 2016;125: 681–692. 10.1016/j.neuroimage.2015.10.089 26541082

[85] Arcaro MJ, McMains SA, Singer BD, Kastner S. Retinotopic organization of human ventral visual cortex. J Neurosci. 2009;29: 10638–10652. 10.1523/JNEUROSCI.2807-09.2009 19710316 PMC2775458

[86] Levy I, Hasson U, Avidan G, Hendler T, Malach R. Center-periphery organization of human object areas. Nat Neurosci. 2001;4: 533–539. 10.1038/87490 11319563

[87] Silson EH, Chan AW-Y, Reynolds RC, Kravitz DJ, Baker CI. A Retinotopic Basis for the Division of High-Level Scene Processing between Lateral and Ventral Human Occipitotemporal Cortex. J Neurosci. 2015;35: 11921–11935. 10.1523/JNEUROSCI.0137-15.2015 26311774 PMC4549403

[88] Silson EH, Groen IIA, Kravitz DJ, Baker CI. Evaluating the correspondence between face-, scene-, and object-selectivity and retinotopic organization within lateral occipitotemporal cortex. J Vis. 2016;16: 1–21. 10.1167/16.6.14 27105060 PMC4898275

[89] Groen IIA, Silson EH, Baker CI. Contributions of low- and high-level properties to neural processing of visual scenes in the human brain. Philos Trans R Soc B. 2017;372: 1–11. 10.1098/rstb.2016.0102 28044013 PMC5206270

[90] Güçlütürk Y, Güçlü U, van Gerven M, van Lier R. Representations of naturalistic stimulus complexity in early and associative visual and auditory cortices. Sci Rep. 2018;8: 3439 10.1038/s41598-018-21636-y 29467495 PMC5821852

[91] Malcolm GL, Groen IIA, Baker CI. Making sense of real-world scenes. Trends Cogn Sci. 2016;20: 843–856. 10.1016/j.tics.2016.09.003 27769727 PMC5125545

[92] Peelen M V, Kastner S. Attention in the real world: toward understanding its neural basis. Trends Cogn Sci. Elsevier Ltd; 2014; 1–9. 10.1016/j.tics.2014.02.004 24630872 PMC4908952

[93] Kaiser D, Oosterhof NN, Peelen M V. The Neural Dynamics of Attentional Selection in Natural Scenes. J Neurosci. 2016;36: 10522–10528. 10.1523/JNEUROSCI.1385-16.2016 27733605 PMC6601932

[94] Malcolm GL, Henderson JM. The effects of target template specificity on visual search in real-world scenes: Evidence from eye movements. J Vis. 2009;9: 1–13. 10.1167/9.11.8 20053071

[95] Groen IIA, Ghebreab S, Lamme VAF, Scholte HS. The role of Weibull statistics in rapid object detection in natural scenes. J Vis. 2010;10: 992 10.1167/10.7.992

[96] Jegou H, Douze M, Schmid C. Hamming embedding and weak geometric consistency for large scale image search. Proceedings of the 10th European conference on Computer Vision. 2008.

[97] Opelt A, Pinz A, Fussenegger M, Auer P. Generic object recognition with boosting. IEEE Trans Pattern Anal Mach Intell. 2004;28: 416–31. 10.1109/TPAMI.2006.54 16526427

[98] Deng J, Dong W, Socher R, Li L-J, Li K, Fei-Fei L. ImageNet: A large-scale hierarchical image database. 2009 IEEE Conf Comput Vis Pattern Recognit. Ieee; 2009; 248–255.

[99] Olmos A, Kingdom FAA. A biologically inspired algorithm for the recovery of shading and reflectance images. Perception. 2004;33: 1463–1473. 10.1068/p5321 15729913

[100] Jahfari S, Ridderinkhof KR, Scholte HS. Spatial frequency information modulates response inhibition and decision-making processes. PLoS One. 2013;8: e76467 10.1371/journal.pone.0076467 24204630 PMC3804599

[101] Jahfari S, Waldorp L, van den Wildenberg WPM, Scholte HS, Ridderinkhof KR, Forstmann BU. Effective connectivity reveals important roles for both the hyperdirect (fronto-subthalamic) and the indirect (fronto-striatal-pallidal) fronto-basal ganglia pathways during response inhibition. J Neurosci. 2011;31: 6891–9. 10.1523/JNEUROSCI.5253-10.2011 21543619 PMC6632844

[102] Jenkinson M, Bannister P, Brady M, Smith S. Improved optimisation for the robust and accurate linear registration and motion correction of brain images. Neuroimage. 2002;17: 825–841. 12377157 10.1016/s1053-8119(02)91132-8

[103] Woolrich MW, Ripley BD, Brady JM, Smith SM. Temporal autocorrelation in univariate linear modeling of fMRI data. Neuroimage. 2001;14: 1370–1386. 10.1006/nimg.2001.0931 11707093

[104] Sidak Z. Rectangular Confidence Regions for the Means of Multivariate Normal Distributions. J Am Stat Assoc. 1967;62: 626–633.

[105] Ludbrook J. On Making Multiple Comparisons in Clinical and Experimental Pharmacology and Physiology. Clin Exp Pharmacol Physiol. 1991;18: 379–392. 10.1111/j.1440-1681.1991.tb01468.x 1914240

[106] Abdi H. The Bonferonni and Šidák corrections for multiple comparisons. Encyclopedia of Measurement and Statistics. 2007 pp. 103–107.

[107] Woolrich MW. Robust group analysis using outlier inference. Neuroimage. 2008;41: 286–301. 10.1016/j.neuroimage.2008.02.042 18407525

[108] Worsley KJ. Statistical analysis of activation images In: Jezzard P, Matthews PM, Smith SM, editors. Functional MRI: An introduction to methods. Oxford University Press; 2001.

[109] Eickhoff SB, Stephan KE, Mohlberg H, Grefkes C, Fink GR, Amunts K, et al A new SPM toolbox for combining probabilistic cytoarchitectonic maps and functional imaging data. Neuroimage. 2005;25: 1325–35. 10.1016/j.neuroimage.2004.12.034 15850749

[110] Perrin F. Spherical splines for scalp potential and current density mapping. Electroencephalogr Clin Neurophysiol. 1989;72: 184–187. 10.1016/0013-4694(89)90180-6 2464490

[111] Ratcliff R, Tuerlinckx F. Estimating parameters of the diffusion model: approaches to dealing with contaminant reaction times and parameter variability. Psychon Bull Rev. 2002;9: 438–481. 12412886 10.3758/bf03196302 PMC2474747

[112] van Ravenzwaaij D, Oberauer K. How to use the diffusion model: parameter recovery of three methods: {EZ}, fast-dm, and {DMAT}. J Math Psychol. 2009;53: 463–473.

[113] Forstmann BU, Dutilh G, Brown S, Neumann J, von Cramon DY, Ridderinkhof KR, et al Striatum and pre-SMA facilitate decision-making under time pressure. Proc Natl Acad Sci. 2008;105: 17538–17542. 10.1073/pnas.0805903105 18981414 PMC2582260

[114] Mulder MJ, Bos D, Weusten JMH, van Belle J, van Dijk SC, Simen P, et al Basic impairments in regulating the speed-accuracy tradeoff predict symptoms of attention-deficit/hyperactivity disorder. Biol Psychiatry. 2010;68: 1114–1119. 10.1016/j.biopsych.2010.07.031 20926067

[115] Gelman A, Carlin JB, Stern HS, Rubin DB. Bayesian data analysis. Chapman & Hall/CRC; 2003.

