# Supplementary figures and images for: Scene complexity modulates degree of feedback activity during object detection in natural scenes
Source: PLoS Comput Biol. 2018 Dec 31;14(12):e1006690. doi: 10.1371/journal.pcbi.1006690 (PMC6329519; doi:10.1371/journal.pcbi.1006690)

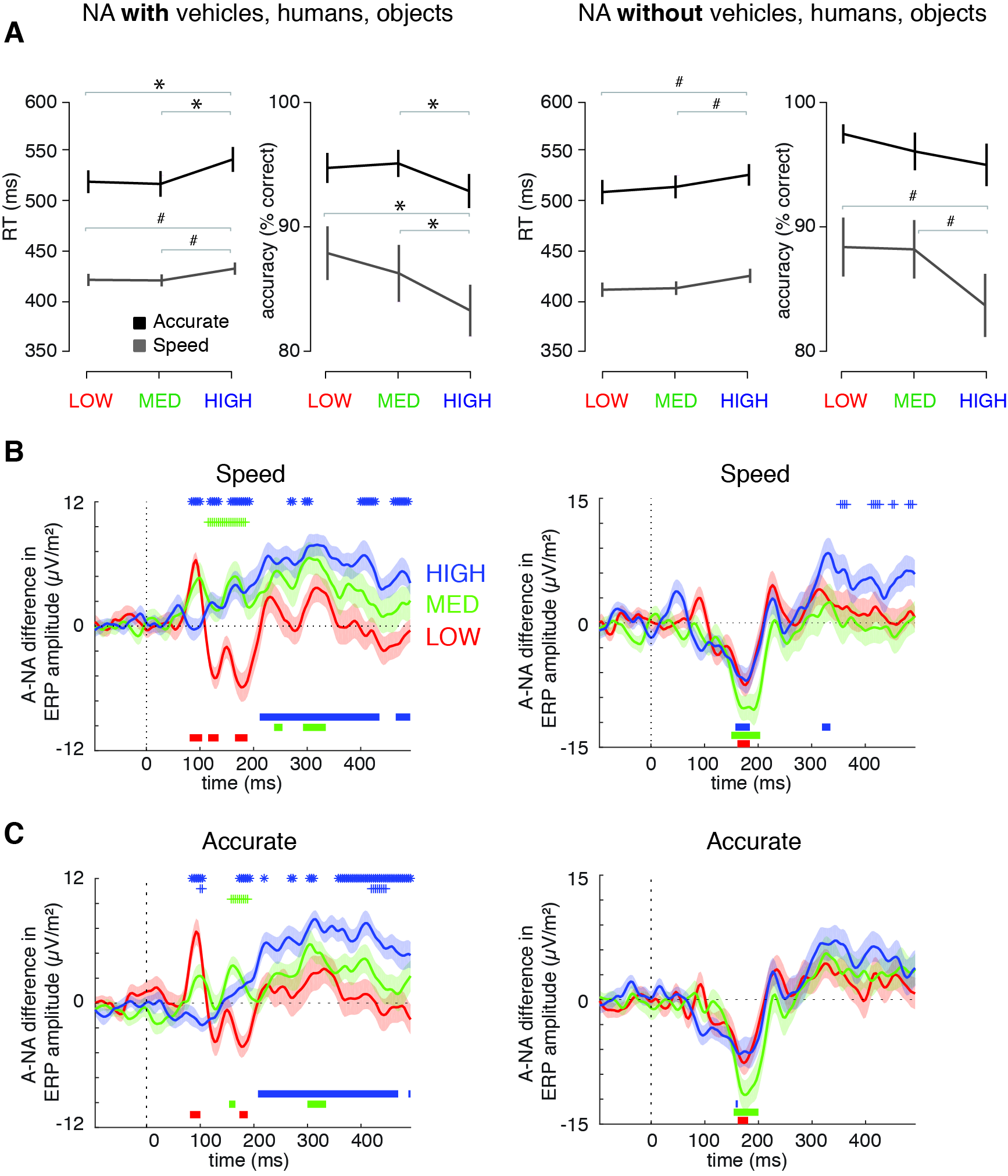

Supplement: S1 Fig — A) Behavioral reaction time and accuracy as a function of complexity (LOW, MED, HIGH) and task instruction (speeded or accurate) when only including (left) or excluding (right) non-animal scenes with vehicles, humans and man-made objects (manually annotated). B-C) Differences in ERP amplitude for animal and non-animal scenes for LOW, MED, and HIGH complexity scenes, computed with (left) or without (right) scenes with vehicles, humans and man-made objects, separately for speed (B) and accurate (C) instructions. The ‘with vehicles, humans, objects’ analysis included 51 non-animal trials for LOW, 58 scenes for MED, and 57 scenes for HIGH; the ‘without vehicles, humans, objects’ analysis included 29 trials for LOW, 22 trials for MED, and 23 trials for HIGH. (TIF) [file pcbi.1006690.s001.tif]
